# Tumour-specific phosphorylation of serine 419 drives alpha-enolase (ENO1) nuclear export in triple negative breast cancer progression

**DOI:** 10.1186/s13578-024-01249-x

**Published:** 2024-06-07

**Authors:** Morgan L Marshall, Kim YC Fung, David A Jans, Kylie M Wagstaff

**Affiliations:** 1 Monash Biomedicine Discovery Institute, Monash University, Clayton, VIC 3800 Australia; 2 Health and Biosecurity, CSIRO, Westmead, NSW 2145 Australia

**Keywords:** Alpha-enolase, Triple negative breast cancer, Protein phosphorylation, Tumour-specific localisation, Nuclear transport

## Abstract

**Background:**

The glycolytic enzyme alpha-enolase is a known biomarker of many cancers and involved in tumorigenic functions unrelated to its key role in glycolysis. Here, we show that expression of alpha-enolase correlates with subcellular localisation and tumorigenic status in the MCF10 triple negative breast cancer isogenic tumour progression model, where non-tumour cells show diffuse nucleocytoplasmic localisation of alpha-enolase, whereas tumorigenic cells show a predominantly cytoplasmic localisation. Alpha-enolase nucleocytoplasmic localisation may be regulated by tumour cell-specific phosphorylation at S419, previously reported in pancreatic cancer.

**Results:**

Here we show ENO1 phosphorylation can also be observed in triple negative breast cancer patient samples and MCF10 tumour progression cell models. Furthermore, prevention of alpha-enolase-S419 phosphorylation by point mutation or a casein kinase-1 specific inhibitor D4476, induced tumour-specific nuclear accumulation of alpha-enolase, implicating S419 phosphorylation and casein kinase-1 in regulating subcellular localisation in tumour cell-specific fashion. Strikingly, alpha-enolase nuclear accumulation was induced in tumour cells by treatment with the specific exportin-1-mediated nuclear export inhibitor Leptomycin B. This suggests that S419 phosphorylation in tumour cells regulates alpha-enolase subcellular localisation by inducing its exportin-1-mediated nuclear export. Finally, as a first step to analyse the functional consequences of increased cytoplasmic alpha-enolase in tumour cells, we determined the alpha-enolase interactome in the absence/presence of D4476 treatment, with results suggesting clear differences with respect to interaction with cytoskeleton regulating proteins.

**Conclusions:**

The results suggest for the first time that tumour-specific S419 phosphorylation may contribute integrally to alpha-enolase cytoplasmic localisation, to facilitate alpha-enolase’s role in modulating cytoskeletal organisation in triple negative breast cancer. This new information may be used for development of triple negative breast cancer specific therapeutics that target alpha-enolase.

**Supplementary Information:**

The online version contains supplementary material available at 10.1186/s13578-024-01249-x.

## Background

Triple Negative Breast Cancer (TNBC) is the most aggressive subtype of breast cancer and is prone to distant metastasis, recurrence post chemotherapy and a short overall survival rate, in comparison to other breast cancers [1]. TNBC is an umbrella term for a heterogenous cohort of breast cancers that are diagnosed by a lack of estrogen (ER) and progesterone (PR) receptor expression and human epidermal growth factor receptor 2 (HER2) amplification. Approximately 10–20% of breast cancers are triple negative [2, 3]. The mainstay for treatment of TNBC is chemotherapy, due to high pathological response rates, yet in a phenomenon known as the “triple negative paradox” [4] the overall prognosis after chemotherapy is reduced. As a result, most patients with metastatic disease die within 3–5 years of diagnosis [1]. Therefore, there is an urgent need to identify novel biomarkers and targetable mechanisms to specifically diagnose and treat TNBC, as chemotherapy has been shown to be ineffective long term and is prone to long lasting and debilitating side effects.

There is a growing amount of evidence recognising the overexpression of alpha-enolase (ENO1) as a biomarker in over 18 types of cancer [5], including TNBC [6–8]. The enolase family of metalloenzymes consists of four isoforms, alpha-, beta- and gamma-enolase and a sperm-specific isoform, enolase 4 [9, 10]. ENO1 is mostly considered to be a 48 kDa glycolytic enzyme responsible for removing water molecules in the conversion of 2-phosphoglycerate to phosphoenolpyruvate as part of the final steps of glycolysis [11]. ENO1 can however be alternatively translated as c-Myc promotor binding protein (MBP-1) [12, 13], a 37 kDa tumour suppressor preferentially expressed under normal glucose and oxygen conditions. However, expression of MBP-1 is downregulated following hypoxic stress [14] along with a concomitant increase in c-Myc regulated ENO1 expression which mediates increased glycolytic activity, this is referred to as the Warburg effect in cancer cells [15, 16].

In many diseases the expression, localisation, and functions of ENO1 are vastly different, it is one of the first identified “moonlighting” proteins [17] and to date is the most differentially expressed protein known, regardless of pathological condition [18]. Despite its alternatively translated isoform playing a tumour suppressive role, expression of ENO1 in breast cancer is reported to contribute to many tumorigenic functions, such as the Warburg effect, invasion and chemoresistance and it is associated with poor prognosis [14, 19, 20]. Although the functions of ENO1 appear to be conserved in many other cancers, most appear to be differentially regulated by alternative mechanisms, such as post-translational modification, mutation of signalling pathways, or tumour-specific protein-protein interactions, across cancer types [21]. In some cancers depleting or inhibiting the expression and activity of ENO1 has been successful in targeting glycolysis, growth, metastasis, and chemoresistance of ENO1 overexpressing tumours, thereby reducing tumorigenicity [16, 19, 20, 22, 23]. Furthermore, studies focussing on immune responses have shown that autoantibodies against ENO1 are a promising prognostic marker in pancreatic cancer [24] and treatment with monoclonal antibodies against ENO1 or DNA vaccination can reduce tumour growth and metastasis in mice [25–27].

In non-tumour cells enzymatically active ENO1 is usually localised in the cytoplasm regulating glycolysis [21], but in tumour cells it supports many other tumorigenic functions, as previously mentioned and reviewed extensively in recent years [17, 27, 28]. In addition to its cytoplasmic localisation ENO1 has been found to be localised on the cell surface [29], in mitochondria [30], and secreted in exosomes [31, 32]. It’s alternatively translated shorter and non-enzymatically active isoform, MBP-1, is usually localised in the nucleus [12] and upregulation of ENO1 mRNA and expression is a negative prognostic marker of distant metastasis-free survival in breast cancer [33]. In epithelial ovarian cancer and invasive ductal carcinoma highly nuclear staining of ENO1/MBP-1 has been shown to be a positive prognostic marker [34, 35]. We propose that characterising the mechanisms that drive the changes in full-length ENO1 protein expression and subcellular localisation in TNBC tumour cell models may lead to novel insights into ENO1 function.

In many types of cancer nucleocytoplasmic transport of proteins is altered [36, 37], and this has serious follow-on effects for processes that regulate tumorigenic functions such as cell cycle, proliferation or metastasis [38]. The mechanisms involved in the regulation of ENO1 intracellular trafficking are poorly understood and potentially un-conserved across pathological conditions. To date, no canonical transport signals have been identified [21, 39, 40] that coordinate ENO1 movement between organelles or that regulate its nuclear export and movement to the cell surface. Some studies have however identified post-translational modifications (PTMs) [41–43], regulation by other proteins [44–46] or long non-coding RNAs [47, 48] as regulators of ENO1 function in various cancers, and some of these also report differential subcellular localisation of ENO1. There are only a small number of studies that focus on establishing the subcellular localisation of ENO1 in cancer in relation to post-translational modifications; where ubiquitination localises ENO1 to the food vacuole of parasites, monomethylation localises ENO1 to the surface of lung cancer cells, and phosphorylation of ENO1 at S282 inhibits its enzymatic activity [21, 42, 49, 50]. We hypothesise that post-translational modification, namely phosphorylation, may be an important regulatory mechanism requiring further study to examine how ENO1 moves in and out of the cell nucleus in cancer. Understanding ENO1 nuclear trafficking in TNBC may be key to understanding its role in tumorigenesis/pathogenesis.

Nuclear transport is a tightly regulated process involving the importin superfamily of nuclear transport proteins [51], members of which mediate protein cargo movement into and out of nuclear pores embedded in the nuclear envelope. Nuclear import is mediated by importins, of which there are multiple a and b isoforms, which recognise a nuclear localisation signal (NLS) in the cargo protein, typically a basic stretch of amino acids. Nuclear export is analogous to this process, whereby export proteins, importin-β homologs called exportins, recognise a nuclear export sequence (NES) in the cargo protein, typically a hydrophobic pattern of amino acids [52]. Nuclear transport is critically required for correct cellular functioning and is therefore a tightly regulated process. Several mechanisms exist in this regard, including regulation of the expression level and distribution of importins and exportins within the cell, and importantly modulation of the target/cargo proteins themselves through post-translational modifications. In particular, phosphorylation of proteins has been identified as a powerful regulator of nuclear transport [53], where post-translational modification of target proteins may alter nuclear transport in three main ways. First, phosphorylation may mask or unmask a NES/NLS, impairing or enhancing importin/exportin recognition. Second, phosphorylation may alter the secondary structure of the protein inducing a conformational change that uncovers or hides the region of the protein containing the NES or NLS. Third, phosphorylation may directly alter the NES/NLS to increase or decrease the affinity/ability of importins or exportins to bind to the target and therefore increase/reduce nuclear transport ability. Considering this, PTMs, including phosphorylation, have been implicated in regulation of ENO1’s subcellular localisation. Where methylation, acetylation and phosphorylation are reported to alter the subcellular localisation and cell surface presentation of ENO1 [40, 42, 54].

Given all of this, the current study characterises the expression and localisation of ENO1 in a TNBC model during tumour progression, identifies a PTM specific mechanism that regulates the tumour-specific cytoplasmic localisation of ENO1 in TNBC cells, and finally identifies potential moonlighting functions of ENO1 in breast cancer that may rely on its tumour-specific nuclear export. To our knowledge, this is the first report of PTM mediated nuclear transport of ENO1 in breast cancer and establishes a key mechanism for future potential therapeutic targeting.

## Results

### Transcription and protein expression of ENO1 is increasingly expressed in MCF10 TNBC progression cell lines

**Fig. 1:**
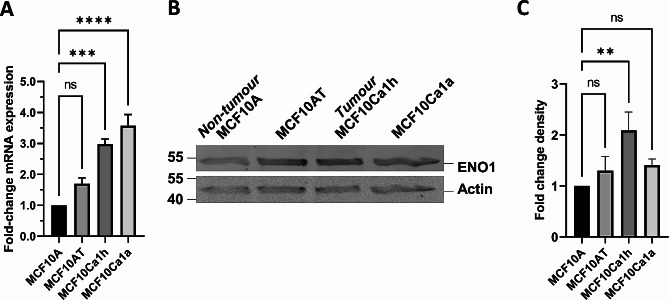
**ENO1 shows increased expression, correlated with the level of tumorigenicity, in MCF10 TNBC cell lines.** (A) Levels of ENO1 mRNA in the MCF10 TNBC tumour progression series of cells as detected by real-time qRT-PCR. ENO1 expression levels in the TNBC tumour progression series of cells, analysed using the -∆∆Ct method. Results represent the mean ± SEM of a single typical experiment from a series of 3 independent biological replicate experiments, presented as fold change relative to the MCF10A normal cells. (B) Western blot of ENO1 expression in MCF10 TNBC cell lines. Cell lysates from MCF10 cell lines were subjected to western blot and probed with the indicated antibodies. ENO1 is shown to expressed in all cell lines with expression increasing in MCF10AT, MCF10Ca1h and MCF10Ca1a in comparison to MCF10A. Actin was used as a loading control. Result representative of a single typical experiment from a series of 3 independent biological replicate experiments. (C) Densitometric analysis of 3 biological replicates of the western blot in panel B, ENO1 expression is normalised to the actin loading controls and represent the fold change increase in density of ENO1 protein in comparison to MCF10A. ** *p* < 0.01, ***p < 0.001, **** *p* < 0.0001 relative to MCF10A, ns – non-significant

Given that ENO1 was previously identified as both a diagnostic and prognostic biomarker in TNBC [6–8, 55], we sought to assess its mRNA transcript levels and protein expression to determine if either correlated with tumour progression in an isogenic model of breast cancer progression. To this end we utilised cells of the MCF10 TNBC tumour progression cell model. The cell lines included in this study were MCF10A – non-tumour, MCF10AT – premalignant, MCF10Ca1h – tumour forming and MCF10Ca1a – metastatic cells. This model is useful for assessing the role of tumorigenicity in biological processes as the cell lines are isogenic, i.e. They are all derived from the same genetic lineage and differ only in their tumorigenic status. mRNA was extracted from each of the cell lines and the samples were analysed by qPCR. Encouragingly, the expression of ENO1 mRNA increased with increasing tumorigenicity of the cells (Fig. 1A). While a trend towards increased expression was observed in the pre-malignant MCF10AT cells (1.6-fold increase compared to MCF10A, non-significant), both MCF10Ca1h and MCF10Ca1a tumour cells demonstrated ~ 3-fold higher ENO1 expression than that observed in the MCF10A normal cells (*p* < 0.001, *p* < 0.0001, respectively). These results indicate that ENO1 expression follows a trend where gene expression increases as the cell becomes fully malignant. To examine whether the increasing trend in mRNA expression observed for ENO1 also results in increased protein expression that is correlated with tumour progression, cell lysates from each of the four TNBC lines were analysed by western blotting (Fig. 1B) and densitometry (Fig. 1C). Protein levels were normalised to Actin as an internal loading control and the relative fold change compared to MCF10A protein expression was calculated. ENO1 protein expression increased by < 1.2-fold in all three transformed cell types, with MCF10a1h cells exhibiting a significant 2-fold increase in protein expression. The protein expression results approximately matched the mRNA expression, where expression increased progressively in the tumorigenic counterparts of the cell model, however we note that protein expression decreases in MCF10Ca1a metastatic cells but does remain increased in comparison to MCF10A non-tumour cells. Only MCF10Ca1h tumour cells showed statistically significant increase in ENO1 protein expression compared to MCF10A non-tumour cells.

### ENO1 subcellular localisation changes dynamically across the MCF10 tumour progression model

**Fig. 2:**
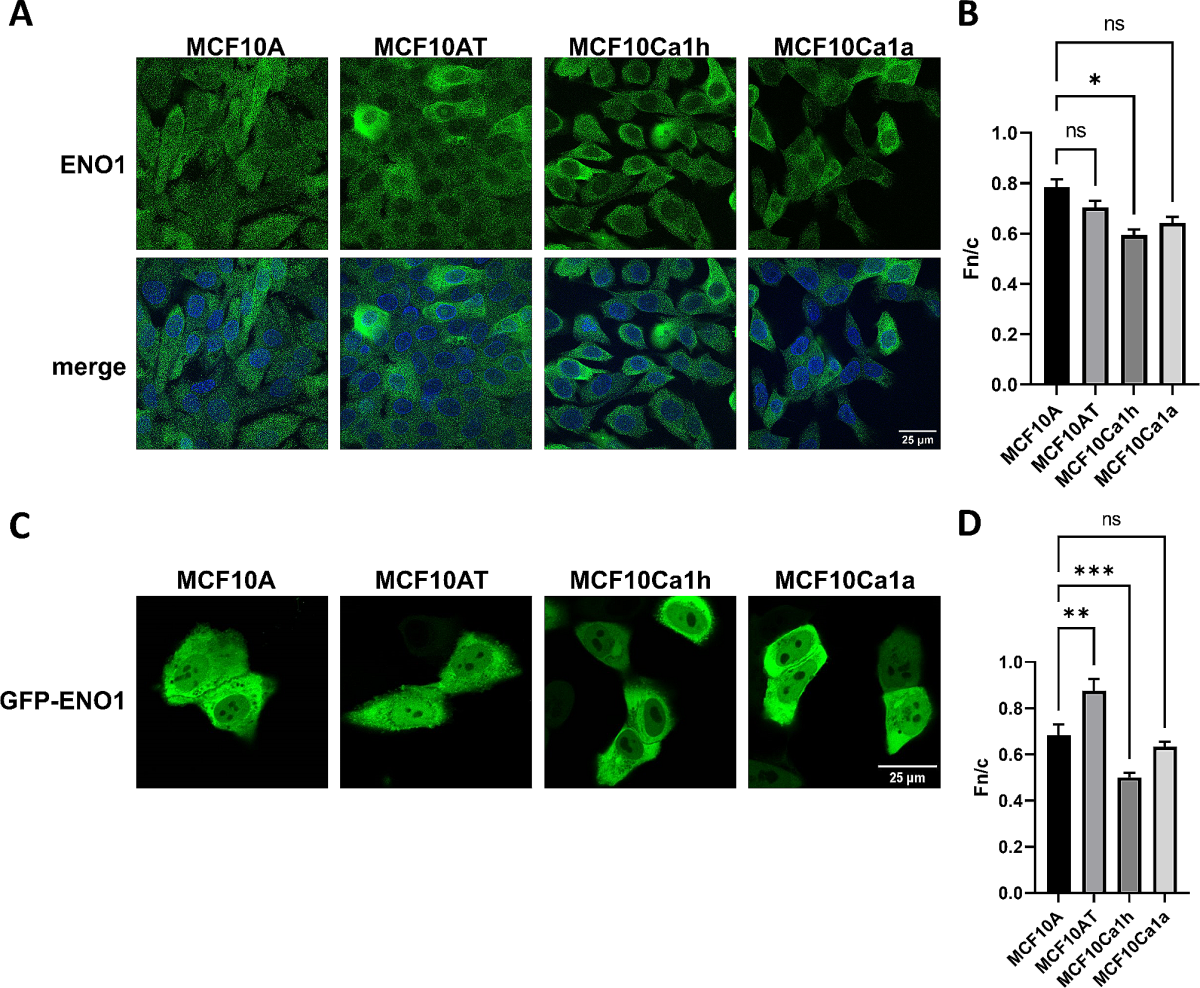
**ENO1 localisation is altered across MCF10 TNBC cell lines.** (A) Typical CLSM images of the indicated MCF10 tumour progression cell lines, fixed and stained using an anti-ENO1 antibody (green) and DAPI to indicate nuclei (blue). (B) Digital images such as those shown in (A) were analysed to determine the nuclear to cytoplasmic fluorescence ratio (Fn/c) (where a ratio below 1 indicates cytoplasmic localisation and above 1 indicates nuclear localisation). Results represent mean ± SEM (*n* > 30) of a single typical experiment from a series of 3 independent biological replicate experiments. (C) Typical CLSM images of the indicated cell lines, transfected to express GFP-ENO1 and imaged live 18–24 h post-transfection. (D) Digital images such as those shown in (C) were analyses to determine the Fn/c as per (B). Results represent mean ± SEM of a single typical experiment from a series of 3 independent biological replicate experiments. * *p* < 0.05, ** *p* < 0.01, *** *p* < 0.001 relative to MCF10A, ns – non-significant

Given that ENO1 expression changes with increased tumorigenicity, we examined whether subcellular localisation of the ENO1 protein was similarly altered in tumour cells using immunofluorescence and confocal laser scanning microscopy (CLSM), specifically to identify any tumour specific trends (Fig. 2A). The digitised images were analysed to assess the specific subcellular localisation by determining the nuclear to cytoplasmic fluorescence ratio (Fn/c; see materials and methods), with an Fn/c > 1 indicating nuclear localisation and an Fn/c < 1 indicating cytoplasmic staining. ENO1 was generally diffuse between the nucleus and cytoplasm in all cell lines (Fn/c between 0.5 and 0.8, Fig. 2B). This was expected as ENO1 is usually reported as a cytoplasmically distributed protein [21]. But interestingly, we observed that the nuclear localisation was significantly reduced in MCF10Ca1h (Fn/c = 0.59, *p* = 0.03) and despite similar reduction in nuclear localisation, MCF10Ca1a (Fn/c = 0.64, *p* > 0.05) was not significantly different to MCF10A (Fn/c = 0.78). Both tumour cell lines showed substantial nuclear exclusion, in comparison to the diffuse nucleocytoplasmic localisation observed in the MCF10A non-tumour cells. Similar to MCF10Ca1a, a non-significant difference was observed in the pre-malignant MCF10AT (Fn/c = 0.7) compared to MCF10A, although an observable trend towards reduced nuclear accumulation was consistently observed. These results suggest that ENO1 nuclear transport is specifically regulated in TNBC cells. To confirm these observations, GFP-tagged ENO1 was transfected into cells of the MCF10 tumour progression model and analysed live 18–24 h later by CLSM, to determine the subcellular localisation of the overexpressed protein. Like that observed for the endogenous protein, GFP-ENO1 was predominately cytoplasmic in all cell lines (Fig. 2C), but significant differences in the extent of nuclear localisation were observed during stages of tumour progression. Unlike the endogenous ENO1 protein, GFP-ENO1 became significantly more nuclear in the MCF10AT cells (Fn/c = 0.87 versus 0.68 in the MCF10A cells, *p* < 0.01; Fig. 2D), but was still predominantly cytoplasmic. In contrast, the MCF10Ca1h cells exhibited a significant decrease in nuclear localisation (Fn/c = 0.50, *p* < 0.001,) compared to MCF10A cells. Intriguingly, no significant difference in localisation between MCF10A and MCF10Ca1a (Fn/c = 0.63, *p* = 0.3) cells was observed. Taken together, these results suggest that ENO1 subcellular localisation is linked to tumour progression. This could be due to increased nuclear export, decreased nuclear import, or increased cytoplasmic retention in tumour cells. Regulation of this mechanism is likely to involve post-translation modification of either ENO1 or its associated nuclear transport factors.

### ENO1 is phosphorylated in TNBC cancer models

**Fig. 3:**
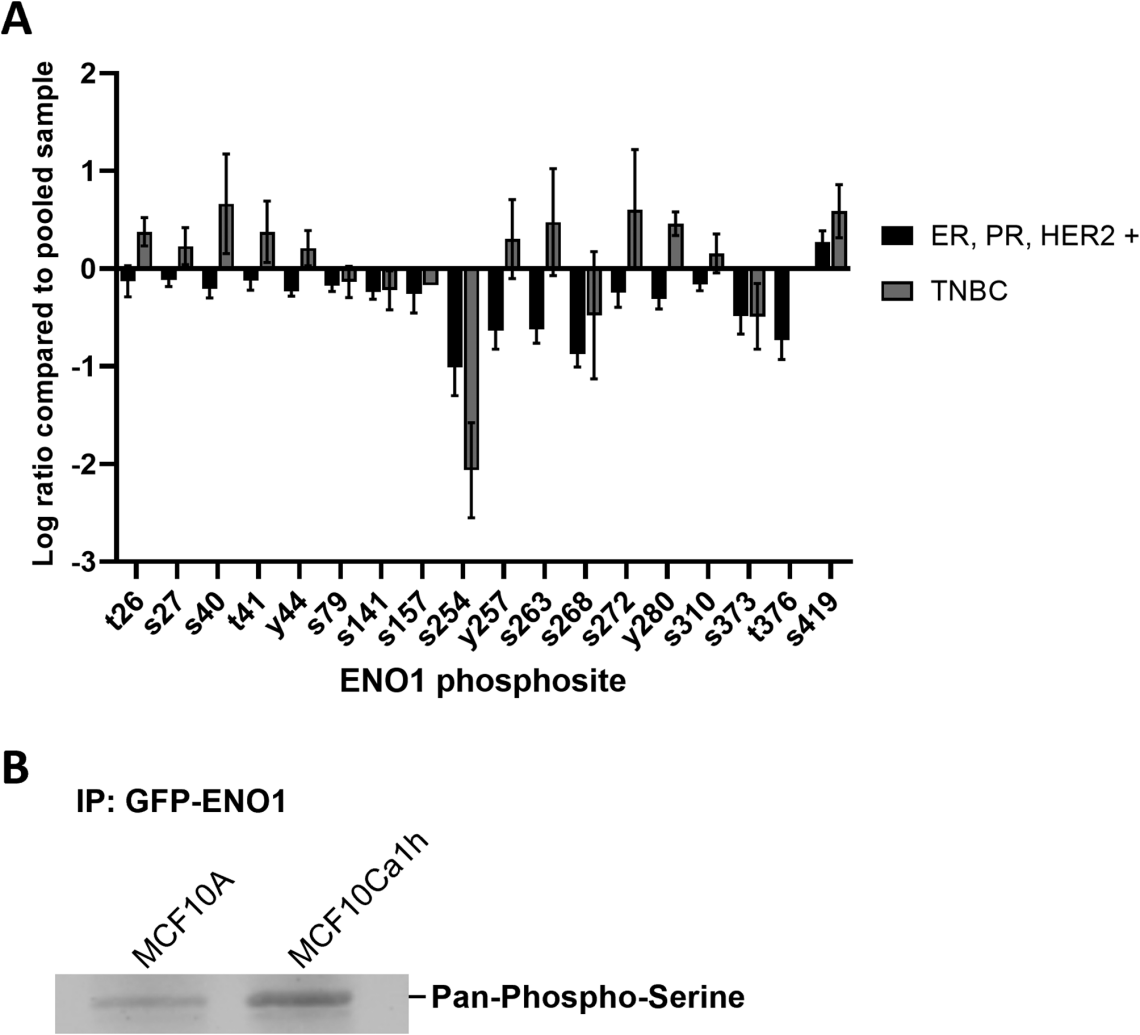
**ENO1 is phosphorylated at S419 in breast cancer patient samples** (A) Data used in this figure was publicly available data generated as stated by the Clinical Proteomic Tumor Analysis Consortium (NCI/NIH). Quantitative mass-spectrometry based phosphoproteomic analyses were performed on breast tumours (125 participants). Data were generated with TMT10plex quantification, and each 10-plex experiment contained a common reference sample composed of a pooled mixture of 40 tumour samples. Data was separated into receptor positive breast cancer (ER, PR, HER2 +) and TNBC manually for the current study, based on reported therapeutic receptor expression. When ER, PR or HER2 status was not stated, samples were assigned to a non-TNBC category. The graph indicates log transformed fold change ENO1 phosphopeptide abundance relative to a pooled breast cancer control sample. (B) Western blot of GFP-ENO1 expression in MCF10A and MCF10Ca1h TNBC cell lines. GFP-trap immunoprecipitations of the indicated MCF10 cell lines were subjected to western blot and probed with anti-phospho-serine antibody. Result representative of a single typical experiment from a series of 3 independent experiments

Despite vast evidence suggesting that phosphorylation is essential for the regulation and function of ENO1 in yeast, parasites and cancer cells, minimal research has been conducted on the effect of these modifications on the proteins subcellular localisation and there is a particular gap in our understanding of ENO1’s nuclear transport in human cells. Considering the multitude of nuclear and cytoplasmic functions and known interactors of ENO1 [21], understanding how ENO1 nuclear transport is regulated is essential for examination of the prognostic and diagnostic value of ENO1 in various cancers. To our knowledge ENO1 phosphorylation has not been examined in breast cancer to date. Given that phosphorylation is a key regulator of nuclear transport mechanisms, and that ENO1 phosphorylation in parasites is correlated with cytoplasmic localisation; where cytosolic isoforms of ENO1, that arise due to phosphorylation, fail to localise the nucleus [40], we examined TCGA phosphomimetic data from breast cancer patient samples to discover ENO1 phosphosites found in TNBC patients [56, 57]. First iTRAQ phosphoproteomic data from breast cancer patient samples was sorted to discern between ER, PR, HER2 positive breast cancers and TNBCs using associated patient histological data. Cases without negative ER, PR, HER2 receptor expression reported were sorted into the receptor positive cohort (Fig. 3A). From this we identified 11 phosphorylation sites on ENO1 present in TNBC samples. Many of these have been identified only in high throughput screens and have no known associated functional outcome (www.phosphosite.org/uniprotAccAction?id=P06733) [58]. Of particular interest was phosphoserine 419 (pS419), which was the only phosphosite expressed in both TNBC and in receptor positive breast cancers, and thus may be a broader marker of breast cancer. For S419 we observed that there was a non-significant increase in phosphorylated S419 abundance in TNBC samples in comparison to receptor-positive samples, suggesting there may be some evidence of significant enhancement of S419 phosphorylation in TNBC patient samples. Novelli and colleagues have previously reported tumour specific enrichment of this phosphosite in pancreatic ductal adenocarcinoma (PDAC), and further showed that this phosphorylation gives rise to acidic isoforms of ENO1 which elicit production of autoantibodies and correlate with better clinical outcomes [24, 41].

Due to its tumour specificity in PDAC and widespread expression in both hormone receptor positive and TNBC patient samples, we examined whether ENO1 was also phosphorylated in our MCF10 cell model. MCF10 cells were transfected with GFP-ENO1 for immunoprecipitation using GFP-trap resin 18–20 h post transfection. Proteins were separated by molecular weight by SDS-PAGE (total protein isolated in immunoprecipitates are shown in Supplementary Figure S1). GFP-ENO1 bands for MCF10 and MCF10Ca1h at approximately 70 kDa were probed with anti-phospho-serine antibodies (Fig. 3B). Both MCF10A and MCF10Ca1h GFP-ENO1 immunoprecipitates were found to show presence of phospho-serine residues by positive staining. The finding of phospho-serine positivity in non-tumour cells is consistent with the result found using 2D-PAGE by Tomaino et al. [41], where only a small amount of the pS419 containing acidic ENO1 isoforms were observed in non-cancer patient samples. Unlike our experiment, Tomaino et al., were able to determine fold change increase in ENO1-pS419 abundance in PDAC patient samples and cell lines, whereas our experiment yielded qualitative results confirming only the phosphorylation status of GFP-ENO1 residues. Furthermore, the presence of serine phosphorylation even in the non-tumour cells, supports our hypothesis that the phenotype displayed in the following experiments is likely an accurate recapitulation of the tumour-specificity of the pS419 modification, in light of the other predicted phospho-serine sites present in ENO1.

### Mutation of S419 alters GFP-ENO1 subcellular localisation

**Fig. 4:**
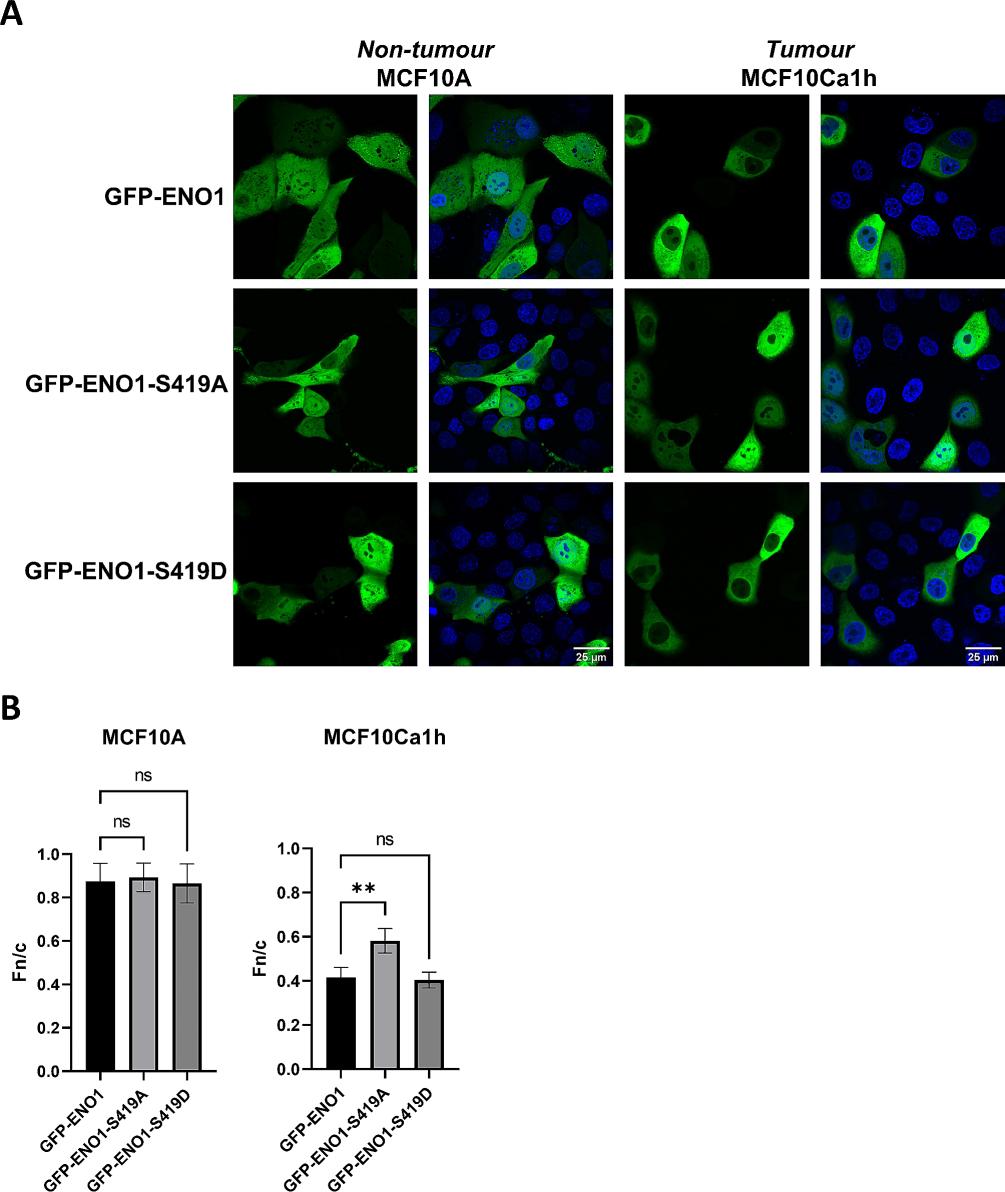
**Phosphomimetic point mutants of ENO1-S419 show altered nuclear transport only in MCF10Ca1h tumour cells.** (A) MCF10A non-tumour and MCF10Ca1h tumour cells were transfected to express GFP-ENO1 and S419 point mutants (S419A phospho-null and S419D phosphomimetic), then analysed by CLSM 18 h later. (B) Images such as those in (A) were analysed to determine the Fn/c ratio as per the legend to Fig. 2. Results represent mean Fn/c ± SEM (*n* > 30) of a single typical experiment from a series of 3 independent biological replicate experiments. ** *p* < 0.01 relative to GFP-ENO1, ns – non-significant. Data was not corrected for multiple comparisons

Given that ENO1 phosphorylation was identified in our cell model, and in multiple other studies [24, 41, 57, 58], we next examined the role of the S419 phosphorylation site in ENO1 subcellular localisation. MCF10A and MCF10Ca1h cells were transfected to express either wild type GFP-ENO1, a GFP-tagged S419A point mutant of ENO1 (GFP-ENO1-S419A) that is incapable of being phosphorylated at this site or the phosphomimetic GFP-ENO1-S419D and imaged live by CLSM (Fig. 4A). No significant differences in localisation of the mutants were observed in MCF10A normal cells, in comparison to wild type GFP-ENO1 (*p* > 0.05; Fig. 4B). Interestingly, in the MCF10Ca1h tumour cells a significant 1.4-fold increase in nuclear localisation was observed for the S419A mutant compared to wild type GFP-ENO1 (Fn/c = 0.41 ± 0.04 vs. 0.58 ± 0.05, *p* = 0.006, Fig. 4B), whereas localisation of the S419D mutant was not significantly different to the wild type (Fn/c = 0.4 ± 0.03 vs. 0.41 ± 0.04, *p* = 0.83). This observed tumour specific nuclear exclusion of the phosphomimetic mutant GFP-ENO1-S419D suggests that positive charge at S419 drives cytoplasmic localisation of ENO1 only in tumour cells, either through reduced nuclear import or more likely through enhanced nuclear export.

### Casein kinase 1 mediated phosphorylation alters nucleocytoplasmic localisation of ENO1

**Fig. 5:**
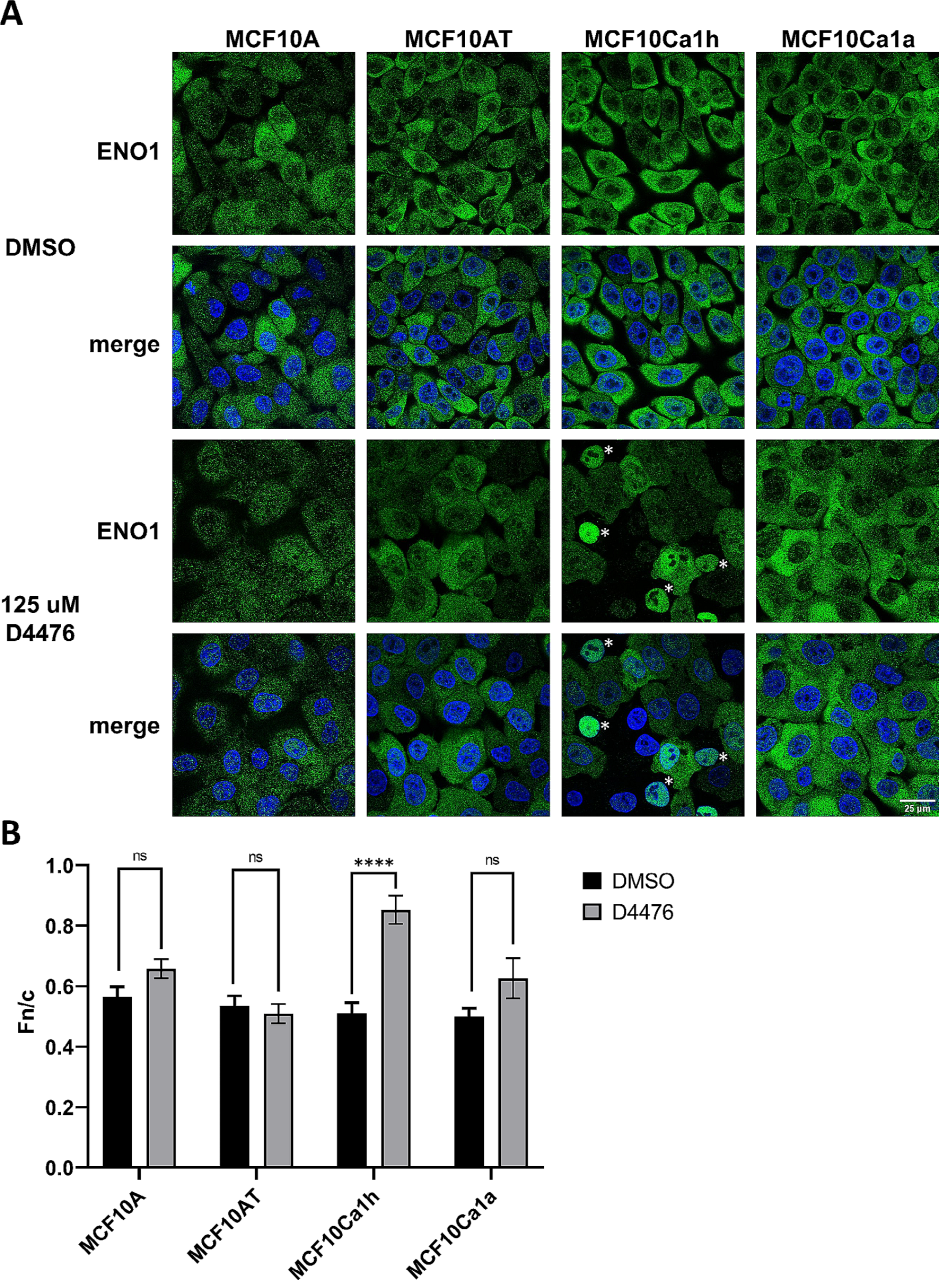
**Casein kinase 1 inhibition with D4476 increases ENO1 nuclear accumulation in MCF10Ca1h tumour cells only.** (A) MCF10 TNBC cell lines that were treated with 125 µM casein kinase 1 inhibitor D4476 or DMSO for 3 h, then fixed and stained with anti-ENO1 antibodies and imaged by CLSM. White star indicates complete nuclear accumulation of ENO1 observed in 10% of D4476 treated MCF10Ca1h cells. Images represent single typical cells from a series of 4 independent biological replicate experiments. (B) Images such as those in (A) were analysed to determine Fn/c ratio as previous. Results represent mean ± SEM (*n* > 50) of a single typical experiment from a series of 4 independent biological replicate experiments. **** *p* < 0.0001 compared to DMSO treated cell line, all other comparisons were nonsignificant. Data was not corrected for multiple comparisons

Given the exciting possibility of a novel tumour specific phosphorylation site in ENO1, we utilised phosphosite prediction software (Phosphomotif Finder: http://www.hprd.org/PhosphoMotif_finder), to predict the Kinase likely to target S419 and determined casein kinase 1 (CK1) to be the most likely candidate. Therefore, we examined whether use of a CK1 specific inhibitor could recapitulate the effect on endogenous ENO1 localisation of the phospho-null GFP-ENO1-S419A mutant. Cells of the MCF10 series treated with 125 μm of the CK1 inhibitor D4476 [62] or DMSO alone. Phospho-null GFP-ENO1-S419A mutant. Cells of the MCF10 series treated with 125 µM of the CK1 inhibitor D4476 [59] or DMSO alone. Cells were fixed and probed with anti-ENO1 antibodies and imaged by CLSM (Fig. 5A). Treatment with D4476 did not alter nucleocytoplasmic localisation of ENO1 in MCF10A, MCF10AT, or surprisingly the fully metastatic MCF10Ca1a cells, however in MCF10Ca1h tumour cells D4476 treatment significantly increased the nuclear accumulation of ENO1 (Fn/c increased from 0.5 ± 0.03 to 0.85 ± 0.04, *p* < 0.0001), Fig. 5B) with ~ 10% of MCF10Ca1h of cells showing a near complete nuclear accumulation of ENO1 (Fig. 5C). To confirm that this increase in nuclear accumulation is specific to inhibition of CK1, MCF10 cell lines were similarly treated with 50 µM of the casein kinase 2 (CK2) specific inhibitor quinalizarin, and then fixed, stained, and imaged by CLSM in the same fashion (Supplementary Figure S2). Quinalizarin treatment did not alter the subcellular localisation of ENO1 in any of the cell lines, demonstrating that CK2 does not play a role in and suggesting that tumour specific nucleocytoplasmic localisation of ENO1 is regulated by CK1 phosphorylation of the S419 site. Interestingly this mechanism does not appear to be active in the metastatic cell line MCF10Ca1a, consistent with the increased nuclear localisation of ENO1 in these cells compared to MCF10Ca1h (Fig. 2).

### S419 phosphorylation enhances nuclear export of ENO1 in TNBC cells

**Fig. 6:**
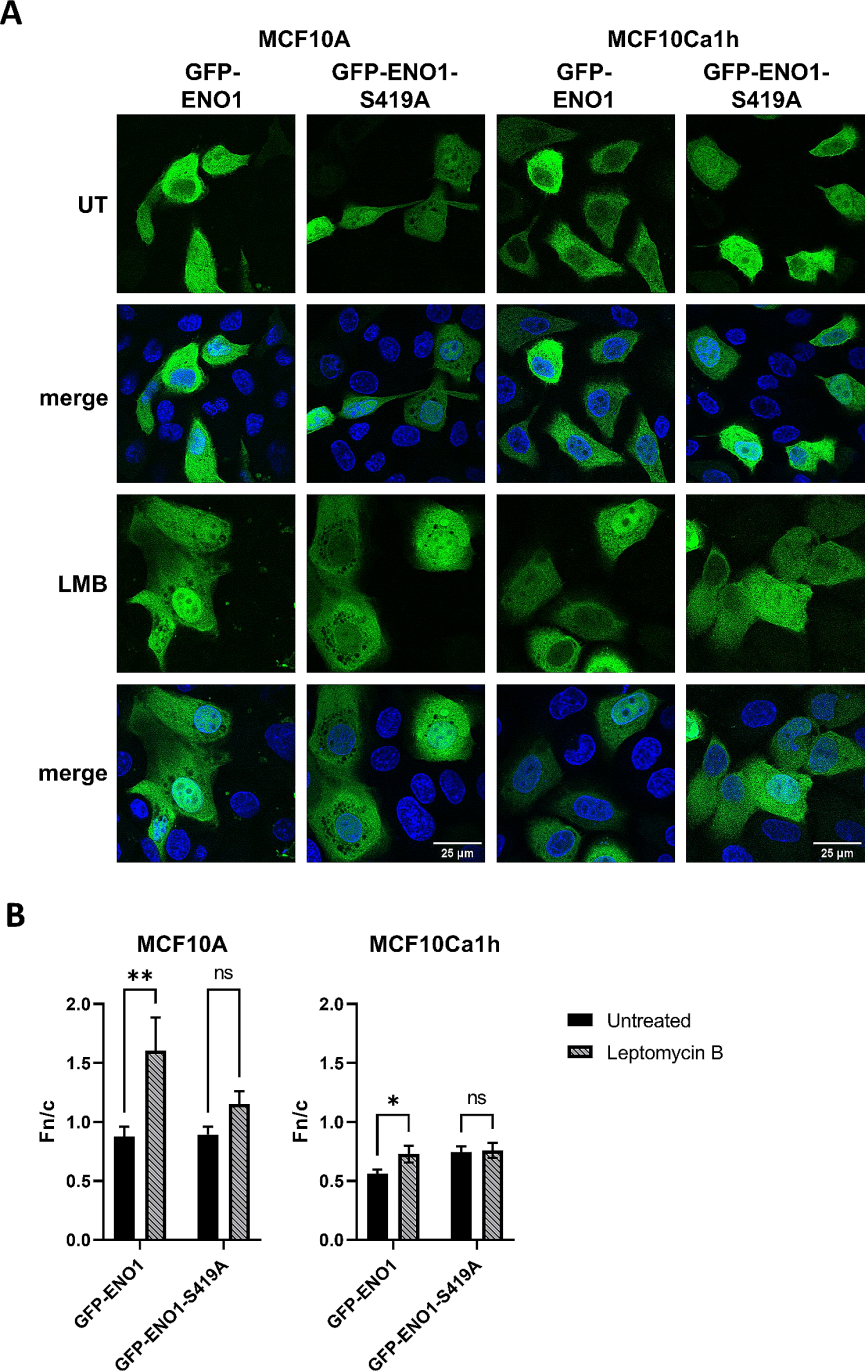
**Inhibition of CRM-1 mediated nuclear export using Leptomycin B (LMB) demonstrates enhanced tumour specific nuclear export of ENO1.** MCF10A non-tumour and MCF10Ca1h tumour cells were transfected with GFP-ENO1 and S419 point mutants (S419A phospho-null and S419D phosphomimetic), treated with LMB or untreated (UT), then fixed and stained with DAPI to define nuclei (blue), and imaged by CLSM. (A) Representative CLSM images of GFP-ENO1 and S419 point mutant transfected MCF10A (left) and MCF10Ca1h (right). (B) Images such as those in (A) were analysed to determine nuclear-cytoplasmic fluorescence ratios (Fn/c). Results represent mean ± SEM (*n* > 30) of a single typical experiment from a series of 4 independent biological replicate experiments. * *p* < 0.05, ** *p* < 0.01, ns – non-significant

Given that inhibition of phosphorylation of ENO1-S419 in the previous experiments resulted in nuclear accumulation of ENO1, it appears that phosphorylation of ENO1-S419 alters its nucleocytoplasmic transport. This could be either through decreased nuclear import or enhanced nuclear export. To determine which, we next examined the effect of blocking global exportin-1 (CRM1) mediated nuclear export on the subcellular localisation of ENO1. To this end MCF10A and MCF10Ca1h cells were transfected to express wild type GFP-ENO1 or GFP-ENO1-S419A, then treated with a specific inhibitor of CRM1 mediated nuclear export; leptomycin B (LMB), fixed and analysed by CLSM (Fig. 6). As previously, no change in localisation was observed for the untreated GFP-ENO1-S419A mutant in comparison to wild type GFP-ENO1 in MCF10A non-tumour cells (Fig. 6A), while a significant increase in nuclear accumulation was observed in MCF10Ca1h tumour cells expressing GFP-ENO1-S419A compared to wild type (Fn/c = 0.56 ± 0.03 vs. 0.7 ± 0.04, *p* = 0.0022). In MCF10A cells LMB treatment significantly increased the nuclear accumulation of both GFP-ENO1 and GFP-ENO1-S419A to similar levels (Fn/c = 1.6 ± 0.27 and 1.1 ± 0.1, respectively, *p* = 0.13; Fig. 6B), indicating that CRM1-mediated nuclear export of ENO1 is active in these cells and that it is not regulated by phosphorylation at S419. In MCF10Ca1h cells, treatment with LMB, significantly increased the nuclear accumulation of wild-type GFP-ENO1 (to Fn/c = 0.72 ± 0.07, *p* = 0.0339), which is importantly the same level of nuclear accumulation as the untreated GFP-ENO1-S419A null point mutant, implying that CRM1-mediated nuclear export is responsible for this difference. Consistent with this, no further increase in nuclear accumulation was observed for GFP-ENO1-S419A in MCF10Ca1h tumour cells following LMB treatment (Fn/c = 0.75 ± 0.06, *p* = 0.8), indicating that inhibition of S419 phosphorylation through mutation or D4476 treatment leads to the same phenotypic response as blocking CRM1-mediated nuclear export. Furthermore, western blot analysis of GFP-tagged ENO1 mutant transfected cell lysates, identical to the above treatments in both MCF10A and MCF10Ca1h, showed no differences in immunoprecipitated phosphorylated (serine-specific) ENO1 following LMB treatment (Supplementary Figure S3). However, we do note that GFP-ENO1-S419A samples in MCF10Ca1h appear to be less bright (indicating lower expression) in comparison to GFP-ENO1-S419D and -WT. Indicating that the loss of nuclear export shown here is CRM1-mediated blockage of nuclear export specific and validly shows the identical phenotype in MCF10Ca1h to loss of phosphorylation and is not an unintended result of LMB treatment altering other interacting cargo (i.e. CK1) activity. Together these results suggest that ENO1 is a nuclear export cargo of CRM1, but that ENO1’s CRM1-mediated nuclear export is regulated in a tumour specific manner by phosphorylation at the S419 site.

### Loss of phosphorylation at S419 does not impact the enzymatic activity of ENO1

**Fig. 7:**
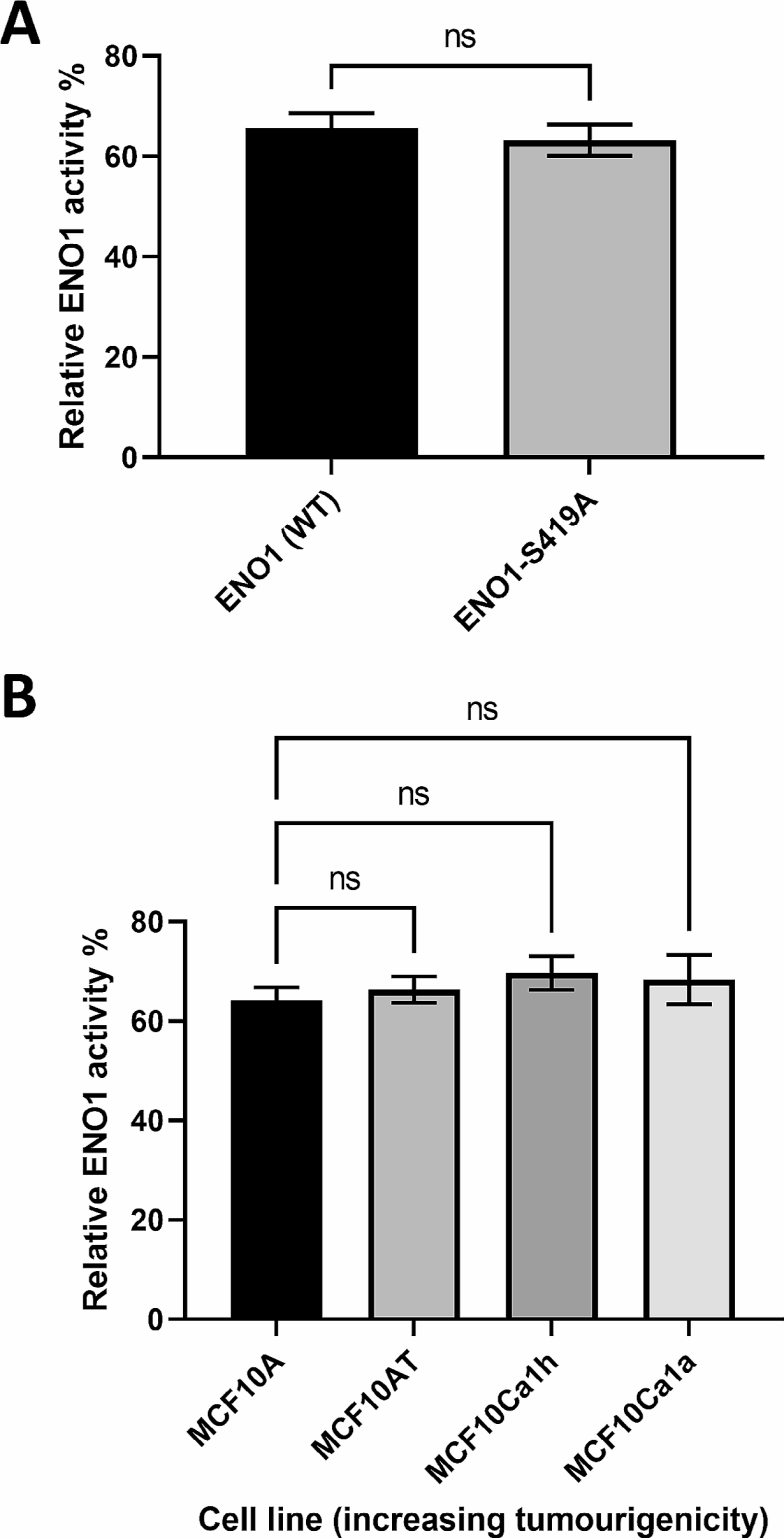
**Relative glycolytic activity of ENO1 is not altered by charge at residue 419, or by tumour progression in MCF10 cell lines.** (A) Recombinant proteins of wild type (WT) full length ENO1 or ENO1-S419A mutant, and equal amounts of cell lysate from MCF10 TNBC cells, were incubated in ENO1 antibody coated 96-well plates, then washed and activity solution containing pyruvate kinase and lactate dehydrogenase added. ENO1 activity was measured by NADH consumption over 60 min. Data is presented relative to the activity of an active ENO1 standard (Abcam, ab89248). (B) Activity of endogenous ENO1 immunopurified from whole cell lysates of the indicated MCF10 TNBC cell lines. Results represent mean ± SEM of a single experiment with 3 biological replicates. ns – non-significant

Protein function is often reliant on PTMs that either regulate activity, localisation, structure, or protein-protein interactions. As ENO1 is an essential glycolytic enzyme that controls the Warburg effect in TNBC tumour cells [28, 33], and its enzymatic activity is known to be downregulated by phosphorylation of ENO1-S282 by unc-51-like kinase 1 in HEK293T cells [50], we examined whether the phospho-null mutation at the S419 phosphorylation site also alters ENO1 enzymatic activity. Potentially, CK1-mediated phosphorylation at S419 may regulate nuclear export of a more active cytoplasmic isoform of ENO1 that promotes increased glycolysis and contributes to tumorigenesis due to its tumour-specific localisation. Recombinant His_6_-tagged wild type ENO1 and phospho-null His_6_-ENO1-S419A proteins were incubated in activity solution containing pyruvate kinase and lactate dehydrogenase, and glycolytic activity measured by tracking NADH consumption over 60 min. Sample activity was made relative to commercially active ENO1 protein (Abcam: ab89248). No significant difference was observed in the relative activity of the wild type (WT) ENO1 (61 ± 7%) and the phospho-null mutant ENO1-S419A protein (64 ± 4%) (Fig. 7A). Next, the relative enzymatic activity of endogenous ENO1 in cells of the MCF10 tumour progression model was examined to determine if ENO1 activity altered in cells correlated with malignant progression (Fig. 7B). We observed that there is no significant difference between the activity of endogenous ENO1 protein captured from any of the MCF10 TNBC cell lysates. This result indicates that the functional outcomes of ENO1-S419 phosphorylation on ENO1 subcellular localisation are likely not to be related to its glycolytic activity.

### Loss of phosphorylation at S419 reduces ENO1 interaction with cytoskeleton regulating proteins

**Fig. 8:**
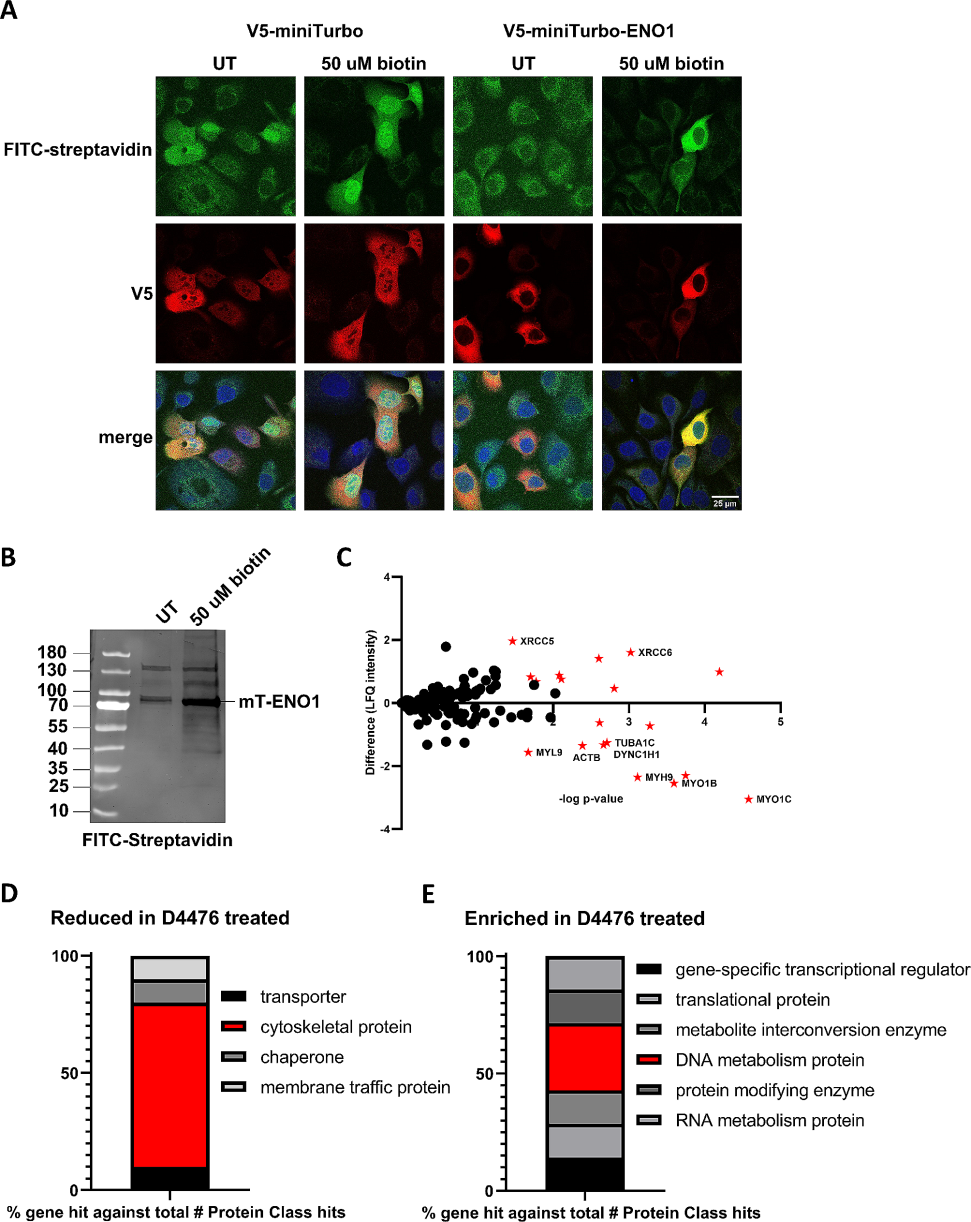
**Inhibited phosphorylation of miniTurbo-ENO1 with CK1 inhibitor D4476 reduces ENO1 interaction with cytoskeletal proteins and increases interaction with DNA-metabolism proteins.** MCF10Ca1h tumour cells were transfected with V5-miniTurbo or V5-miniTurbo-ENO1, then biotinylated by addition of exogenous biotin for 20 min to induce proximity labelling of ENO1 interacting proteins. (A) CLSM images of MCF10Ca1h cells transfected with V5-miniTurbo (left) or V5-miniTurbo-ENO1 (right), then left untreated (UT) or treated with 50 µM biotin, cells were then fixed and stained with anti-V5 antibodies (red), or FITC-streptavidin (green) and nuclei were counterstained with DAPI (blue). Images represent single typical cells from a series of 3 independent biological replicate experiments. (B) Western blot of MCF10Ca1h cell lysates expressing miniTurbo-ENO1 (mT-ENO1) that were either untreated (UT) or treated with 50 µM biotin to induce proximity labelling of other proteins interacting with mT-ENO1. (C) Volcano plot of proteins that showed differences in label free quantitation (LFQ) of expression between MCF10Ca1h cells expressing MiniTurbo-ENO1 that were D4476 treated or DMSO treated, identified by LC-MS. Results represent difference in mean LFQ intensity (D4476 treated – DMSO treated) from 3 replicates, plotted against -log transformed p-values. Red stars indicate statistically significant differences in LFQ intensity between samples with gene name listed. (D) Graphical representation of protein classes present in significantly reduced miniTurbo-ENO1 with D4476 treatment interactor list. Results represent percentage of genes mapped to each protein class listed from *n* = 10 genes. Highest percentage group was coloured red. (E) Graphical representation of protein classes present in significantly enriched miniTurbo-ENO1 with D4476 treatment interactor list. Results represent percentage of genes mapped to each protein class listed from *n* = 9 genes. Highest percentage group was coloured red. Protein classes were assigned using Panther Gene Ontology (GO) analysis online tool. LFQ intensity differences and p-values are reported for all differing proteins in Supplementary Table 1

It is well known that many of ENO1’s non-glycolytic activities stem from interaction with proteins and pathways that regulate functions in cancer such as growth, metastasis, and cellular stress responses [21]. To elucidate the functional outcome of CK1 mediated phosphorylation of ENO1, we employed biotin ligase mediated proximity labelling (miniTurboID) [60] V5-tagged miniTurbo and V5-tagged miniTurbo-ENO1 were expressed in MCF10Ca1h cells. V5-miniTurbo-ENO1 maintained a cytoplasmic localisation in comparison to V5-miniTurbo alone, which had a spread nucleocytoplasmic localisation as expected (Fig. 8A, red panels). It is important to note that expression of miniTurbo alone resulted in a significant highly nuclear background biotinylation, and expression of mini-Turbo-ENO1 in cells that were not biotin treated showed presence of endogenously biotinylated mitochondrial proteins (based on the distribution profile) when probed with FITC-tagged streptavidin (Fig. 8A, green panels). Therefore, the proximity labelling experiment was additionally controlled by adding in matched samples that were not treated with biotin to control for background biotinylation that may confound true interactors. To determine the effect of CK1 mediated phosphorylation on the function of ENO1, MCF10Ca1h cells expressing miniTurboID-ENO1 were treated with 125 µM D4476 or DMSO as a negative control. These cells were then treated with biotin for 20 min at 37 °C to induce proximity labelling of interacting proteins. Cell lysates were then analysed by western blot using anti-biotin antibody. The results (Fig. 8B) show increased biotinylation of proteins in the whole cell lysate after biotin treatment. A substantial increase in biotinylated miniTurbo-ENO1 (mT-ENO1) signal was observed in biotin treated samples, likely due to the propensity of ENO1 to homodimerise and therefore biotinylate each other. To isolate the biotinylated miniTurboID-ENO1 interacting proteins, treated cell lysates were immunoprecipitated using magnetic streptavidin beads in triplicate and then the proteins were digested on bead prior to analysis by LC-MS/MS. After subtracting background proteins and endogenously biotinylated carboxylase proteins [61] found also in the control samples, 151 proteins showed differences in the relative amount of biotinylated protein between DMSO and D4476 treated samples (Supplementary Table 1), with 19 of these being significantly increased or decreased in D4476 treated versus DMSO (Fig. 8C, red stars, named proteins signify majority protein classes affected; DNA repair & cytoskeletal myosin, actin and tubulin). It is worth noting that importin-α2 was significantly downregulated following D4476 treatment, suggesting a possible additional role of reduced nuclear import in the regulation of ENO1 subcellular localisation. After subjecting the lists of significantly enriched and significantly decreased proteins to Gene Ontology (GO) analysis of biological functions, it was observed that myosin heavy chain binding, microfilament motor activity, calmodulin binding, and ATP binding were all significantly downregulated in D4476 treated samples (Fig. 8D - grouped by protein class). Suggesting that wild type phosphorylated ENO1 is involved in microtubule organisation functions through interactions with myosin, tubulin, dynein, and actin proteins. This result further supports ENO1’s role in invasion and metastasis in TNBC. In contrast, biological functions such as DNA repair, nuclear telomere capping, interaction with aggresomes, and ribonucleoprotein complexes were upregulated in the D4476 treated non-phosphorylated sample (Fig. 8E). The shift of non-CK1-phosphorylated ENO1 to interact with DNA-repair, telomeric capping complexes and ribonuclear protein complexes is interesting and supports ENO1’s role as a molecular chaperone and may be representative of the non-glycolytic functions that ENO1 mediates in non-tumour cells.

## Discussion

ENO1 has previously been suggested to be a biomarker of TNBC and in many cancers ENO1 is known to be a diagnostic or prognostic marker. Some studies have shown that ENO1 expression is regulated by well-known oncogenic signalling pathways in TNBC [19] and that it contributes to functions such as proliferation and invasion in breast cancer [33, 62]. In this study we examine the diagnostic capacity of ENO1 expression during TNBC tumour progression. We, like the research of others [20, 33], found that ENO1 mRNA and protein expression is increased in breast cancer tumour cells. By using the genetically identical MCF10 TNBC tumour progression cell model we determined that ENO1 expression increases progressively during TNBC tumour progression and furthermore we noted that the subcellular localisation of ENO1 also dynamically changed during tumour progression. Specifically, we show that ENO1 becomes highly cytoplasmic and excluded from the nucleus of MCF10Ca1h tumour cells in comparison to a more spread localisation in MCF10A non-tumour cells. Consistent with our results, a study by Czogalla et al. [34] found that subcellular localisation of ENO1/MBP-1 in epithelial ovarian cancer patient samples was a prognostic marker, where highly nuclear ENO1/MBP-1 was associated with lower tumour grade and higher overall survival. A point of difference is that the Czogalla study did not discern between expression of full length ENO1 or its shortened isoform MBP-1 and assume all nuclear ENO1 is MBP-1. Further analysis shows the antibody used for that study recognises an epitope in the first 20 amino acids of the protein and therefore could not recognise MBP-1 which lacks the first 90–93 amino acids of the full length ENO1 protein. Indicating that nuclear localisation of full length ENO1, rather than a combination of ENO1 and MBP-1, may be a prognostic maker for epithelial ovarian cancer and supports our result that cytoplasmic localisation of full length ENO1 expression may be related to malignant transformation/tumour progression in TNBC.

PTMs affect the subcellular localisation and subsequent tumorigenic functions of ENO1 in lung cancer, where methylation of ENO1-R50 mediates cell surface trafficking of ENO1 and amino acid substitution (R50K) reduced the invasive capacity of A549 cells in comparison to wild type ENO1 [42]. Furthermore, increasing evidence suggests that ENO1s function is also linked to subcellular localisation [21, 63]. We have confirmed expression of the tumour-specific pS419 ENO1 isoform in TNBC patient samples using TCGA data and have found supportive results for ENO1 being phosphorylated in MCF10 TNBC cell lines. This PTM was first reported in pancreatic ductal adenocarcinoma as a positive prognostic and diagnostic marker, and the presence of phosphorylated ENO1 isoforms was sufficient to produce an in vivo humoral response in PDAC patients [24, 41]. We determined that CK1 mediates phosphorylation of S419, and this plays a significant role in ENO1’s movement between the nucleus and cytoplasm, and defects in subcellular localisation following phospho-inhibition with CK1 inhibitor D4476 can be recapitulated by a serine to alanine point mutation at residue 419. Furthermore, both point mutation and inhibition using D4476 mimic the effects of blocking CRM1-mediated nuclear export of ENO1 in a tumour specific manner. We did note that there seemed to be no effect of D4476 or LMB treatment on ENO1 localisation in the more severe MCF10Ca1a cell line. This was unexpected but suggests that this metastatic cell line could be more genomically unstable than that of the less invasive MCF10Ca1h tumour cell line, and that perhaps the signalling necessary to see this effect no longer exists in the metastatic cell line. It is important to note that D4476 also is a competitive inhibitor of ALK5 and p38α MAP kinase, furthermore widespread CK1 inhibition may also impact the interactions of other proteins with ENO1 or their associated functions, partially explaining the loss of MCF10Ca1a response as above, suggesting that the effect we see is not a direct on/off switch for ENO1 nuclear transport, but instead a key regulator in a larger signalling pathway. This fact however does not diminish our finding, encouragingly, studies have shown that D4476 induced CK1 inhibition sensitises colorectal cancer cells to 5-fluorouracil via inhibition of autophagy pathways [64]. This suggests a role in more than just regulating protein nuclear transport, and that the widespread reach of CK1 signalling may represent a broad substrate inhibitor for a range of tumorigenic functions.

To support the results of the ENO1-S419 phosphomimetic and phospho-null changes in localisation further experiments confirming CK1 as the direct kinase acting on the S419 residue using radioactive kinase assays or alike; and investigation of upstream and downstream signalling pathways affected by D4476 treatment should be assayed, which would be achieved most simply by western blotting. Further experiments could also examine a broader range of cellular functions that ENO1 is reported to be involved in such as metabolism, cell cycle, tumour cell mobility, and drug resistance following D4476 treatment.

**Fig. 9:**
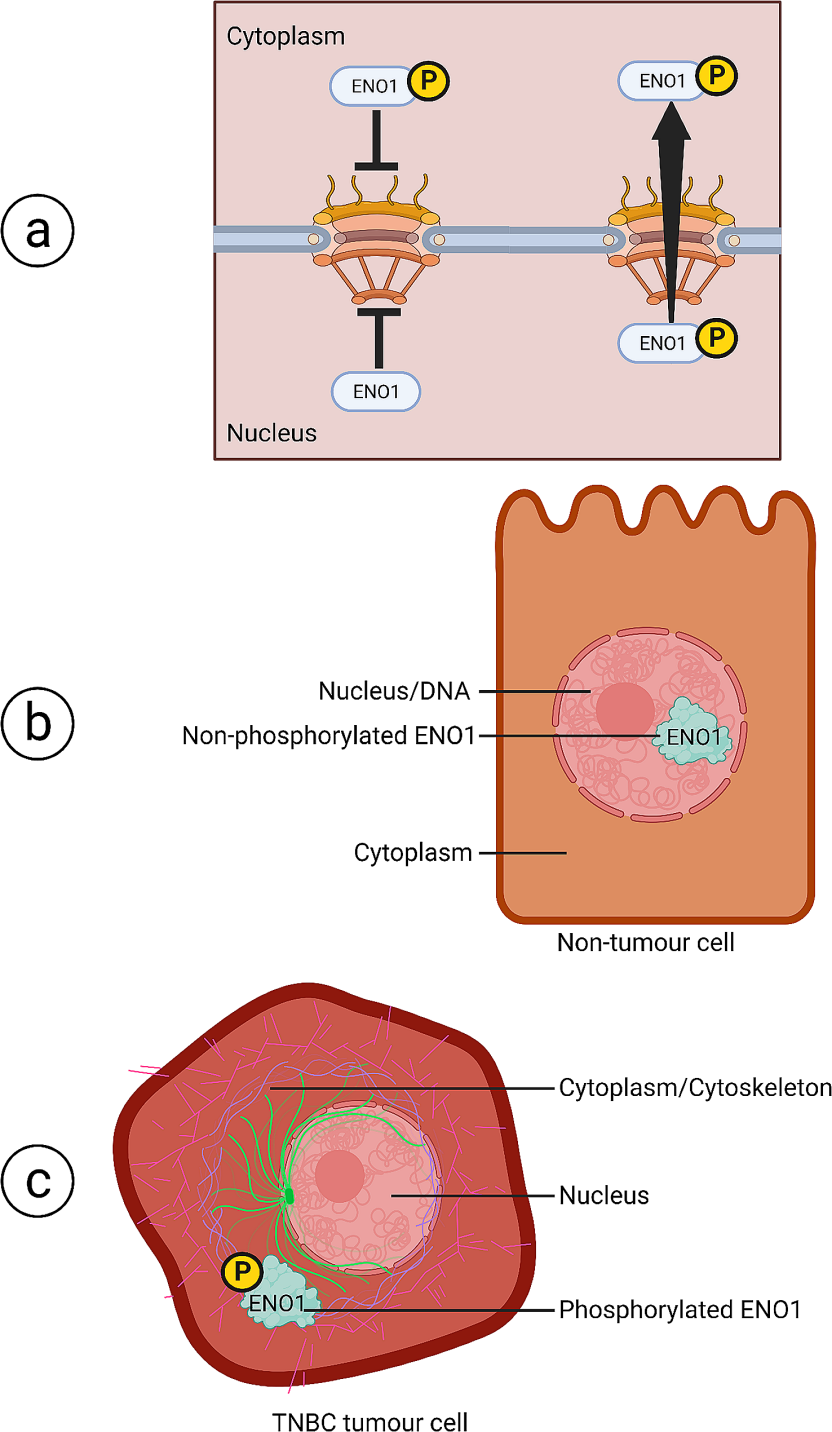
**Proposed model of phosphorylated ENO1’s nucleocytoplasmic transport and associated roles in non-tumour MCF10A and tumorigenic MCF10Ca1h cells.** (A) Diagram of proposed model of ENO1-S419 phosphorylation-mediated regulation of tumour-specific nucleocytoplasmic transport. Upon ENO1-S419 phosphorylation we suggest that ENO1 is unable to enter the nucleus, whereas a lack of S419 phosphorylation renders ENO1 unable to be exported to the cytoplasm (left side). Our results may also suggest that phosphorylated ENO1-S419 specifically exhibits enhanced nuclear export in comparison to that observed in non-tumour cells (right side). (B) In non-tumour cells non-phosphorylated ENO1-S419 shows increased localisation in the nucleus, possibly supporting functions such as DNA-repair or transcriptional regulation. (C) In tumour cells phosphorylated ENO1-S419 is mostly excluded from the nucleus and localised in the cytoplasm, possibly supporting functions such as cytoskeletal organisation or molecular chaperoning of proteins to the cell membrane when phosphorylated. Figure created on Biorender.com

It is commonly reported that ENO1 contains no canonical subcellular localisation signals [65]. Consistent with this, we could find no studies that link nucleocytoplasmic localisation of ENO1 with classically defined nuclear transport mechanisms that rely on nuclear localisation and export signals. The results of this study suggest a model whereby PTM’s regulate the nuclear transport of ENO1 and its subsequent subcellular localisation and ultimately its functions in TNBC. Aspects of this are described in Fig. 9, where our model suggests that nuclear transport complexity is significantly increased by tumour-specific phosphorylation of ENO1 in TNBC cells, where a phosphorylation may regulate ENO1 nuclear transport in a number of ways, either by phosphorylation inhibiting nuclear import, or loss of phosphorylation inhibiting nuclear export. Alternatively, we also suggest that phosphorylation of ENO1 may instead enhance nuclear export resulting in cytoplasmic localisation in tumour cells (Fig. 9a, described graphically). Our results have shown that null point mutation of ENO1-S419, recapitulating the effect of non-phosphorylation of this site, is essentially phenotypically identical to the effect of blocking CRM1-mediated nuclear export. Thus, suggesting that in tumour cells S419 phosphorylation plays a role in the regulation/enhancement of CRM1-mediated nuclear export of ENO1. To this end our study identifies a novel TNBC tumour specific nuclear export mechanism utilised by ENO1. Similar studies have observed phosphorylation mediated nuclear export as tumour specific regulator for the viral protein apoptin [66]. Where apoptin contains a CRM1 recognised nuclear export signal that is turned off in transformed human osteosarcoma cells following phosphorylation [67]. Our study was unable to identify a functioning nuclear export signal, even after investigating four possible candidates (unpublished data), but the point remains that ENO1-S419 phosphorylation appears to play a role in the recognition or regulation of CRM1/ENO1 interaction. Further study of the exact mechanisms behind this interaction will be of great use in nuclear transport inhibitor drug development or may simply shed light on the TNBC specificity of this mechanism, whereby tumour specific nuclear transport of ENO1 via S419 phosphorylation may function in a variety of cancers or other non-tumour tissue types as a functional adaptation.

Finally, we show that the functional outcome of ENO1-S419 phosphorylation may not be linked to the glycolytic activity of the enzyme, unlike other sites such as S282, which when phosphorylated showed decreased glycolytic activity in mammalian cell culture models [50]. This assay ruled out metabolic increase/the Warburg effect as the main function mediated by ENO1 phosphorylation in TNBC. Simpler assays such as cell invasion and proliferation would ideally have been used, however our ENO1-S419 point mutant constructs caused cell death when allowed to express for more than 24 h in MCF10A non-tumour cells (Supplementary Figure S4). This did not allow us to test the tumorigenic functions of ENO1 in our TNBC cell model as these types of assays typically run over several days to weeks. Instead using miniTurboID based proximity labelling of ENO1’s interactors in MCF10Ca1h tumour cells, we have shown that CK1 mediated phosphorylation of ENO1 may be involved in ENO1’s interaction with cytoskeletal proteins such as myosin, tubulin, and actin (localisation depicted in Fig. 9c). Whereas CK1 inhibition using D4476 suggests that non-phosphorylated ENO1 interacts with proteins that have functions in DNA-repair and telomere capping within the nucleus (localisation depicted in Fig. 9b). These results suggest that CK1 phosphorylated ENO1 may have roles in cytoskeletal remodelling, tumour cell invasion and migration, whereas the dephosphorylated form in non-tumour cells plays roles in DNA-repair and telomere capping that may either contribute to or inhibit cancer related genomic instability. It will be important to validate these results using other methods such as co-immunoprecipitation and western blotting, or immunofluorescent colocalisation and CLSM imaging. This is to control for the possible limitations of miniTurboID proximity labelling where promiscuous off-target biotinylation may occur for non-target interactors or mass spectrometry results may be confounded by abundant endogenously biotinylated proteins, despite our best efforts designing appropriate controls and excluding known abundant endogenously biotinylated proteins.

Considering we observed the localisation of the non-phosphorylated and phospho-null mutant to be like that of wild type ENO1 in non-tumour cells we hypothesise that, like in ovarian cancer, nuclear ENO1 may be a positive prognostic marker and is likely to be playing protective roles perhaps in DNA-repair or may simply function redundantly similar to its shorter isoform MBP-1 and have a role in transcriptional regulation until phosphorylated at S419 in tumour cells. Identifying the functions regulated by CK1 mediated phosphorylation of ENO1, is an important step in the future development of therapeutic strategies to treat ENO1 expressing TNBC. Some studies to date have suggested CK1 as a therapeutic target for TNBCs [68]. Bar and colleagues have shown that silencing of casein kinase 1 delta reduced proliferation, invasion, and migration of MDA-MB-231 TNBC cells and reduced lung metastasis of breast cancer xenografts in mice [69]. Some non-breast cancer studies have also shown that CK1 is a direct regulator of Wnt/β-catenin signalling in mouse skin carcinogenesis, where stabilisation of CK1 activates Wnt target genes, and similarly CK1 inhibition results in downregulation of Wnt target genes. Another study shows similar roles to that observed in TNBC in epithelial ovarian cancer [70], indicating that the functions of CK1 in cancer show conservation through other tumour types. Our results compliment these studies by suggesting a downstream target of CK1, which with further study may be able to link both ENO1-S419 phosphorylation and aberrant CK1 expression in a panel of both diagnostic and prognostic biomarkers for TNBC.

The use of ENO1-S419 phosphorylation as a biomarker requires further quantitative investigation to determine if expression of this phosphorylated isoform is different between normal and tumorigenic cells, however we are confident that the experiments in this study assure tumour specific action in breast cancer despite phosphorylated ENO1 being present in non-tumour MCF10A cell lines as our research shows there are functional differences based on localisation and protein-protein interactions. We suggest that subcellular localisation and ENO1-S419 phosphorylation may be a useful measure of TNBC tumorigenicity, where malignant breast cancer cells likely show highly cytoplasmic localisation of ENO1, whereas normal breast cancer cells would be likely to show spread nucleocytoplasmic localisation of ENO1. Altogether this study provides a step forward in the understanding of the moonlighting protein and cancer biomarker ENO1 during TNBC tumour progression and suggests a targetable mechanism for development of diagnostic and prognostic screening of TNBC based on phosphorylation status of ENO1.

## Methods

### Antibodies, reagents, and plasmids

Anti-actin (3700) and anti-α/β tubulin (2148) antibodies were purchased from Cell Signaling Technology. Anti-ENO1 (ab155102) and pan-phospho-serine (ab9332) antibodies were purchased from Abcam. Anti-V5 (R960-25) antibody and streptavidin-FITC conjugate (S-869) were purchased from Thermo Fisher Scientific. Anti-GFP (11,814,460,001) antibody was purchased from Sigma-Aldrich. Li-Cor IRDYe fluorescent secondary antibodies 680-anti-rabbit (926-68071) and 800-anti-mouse (926-32210) were purchased from Millennium Science. D4476 casein kinase I inhibitor was purchased from Abcam. Leptomycin B was purchased from BioAustralis. Quinalizarin, protease inhibitor cocktail, phosStop inhibitor and other chemicals were purchased from Sigma-Aldrich, or as otherwise specified. The plasmids encoding GFP-ENO1, GFP-ENO1-S419A and GFP-ENO1-S419D were generated by GenScript in pcDNA 3.1-N-eGFP. Plasmids encoding V5-miniTurbo-NES were obtained from Professor Mike Ryan (Monash University), mutagenesis resulting in removal of the NES sequence and insertion of ENO1’s sequence was performed by GenScript.

### Cell culture and treatments

The isogenic TNBC tumour progression model of human breast epithelial cell lines MCF10A, MCF10AT, MCF10Ca1h & MCF10Ca1a [71] were cultured as previously [72] in DMEM/F12 medium with 10 mM HEPES, supplemented with 2.2 g sodium bicarbonate, 5% horse serum, 100 ng/ml cholera toxin, 0.5 µg/ml hydrocortisone, 10 µg/ml bovine insulin and 20 ng/ml human epidermal growth factor. Cells were not used past 20 passages to avoid spontaneous tumour progression.

For transient overexpression of GFP-tagged ENO1 constructs, cells were pre-seeded onto glass coverslips (24 mm round or 15 cm square), fluorodishes, 8-well ibidi microslides (Ibidi, DKSH), or in 10 cm culture dishes, then transfected with GFP-ENO1, -S419A or -S419D plasmid DNA using Lipofectamine 3000 (Thermo Fisher) according to the manufacturer’s instructions. Cells were imaged live 18–22 h post-transfection, fixed as below for imaging, or collected for immunoprecipitation. Immunocomplexes were precipitated using GFP-Trap resin (ChromoTek) according to manufacturer instructions.

For drug treatments with kinase inhibitors, cells were pre-seeded onto glass coverslips (15 cm square) or 8-well ibidi microslides (Ibidi, DKSH) then treated with D4476 (125 µM) or Quinalizarin (50 µM) for 3–4 h at 37 °C. Dimethylsulfoxide (DMSO) was used as the drug vehicle and was administered in untreated cells as a control in equal quantities. Kinase inhibitor concentrations were chosen as per manufacturers recommendations.

For miniTurboID experiments, cells were pre-seeded in 15 cm dishes, then transfected with V5-miniTurbo or V5-miniTurbo-ENO1 plasmid DNA using Lipofectamine as above. 16–20 h post-transfection, dead cells were washed away with PBS, then cells were treated with DMSO or D4476 (125 µM) for 3 h at 37 °C. Cells were washed again and treated with 50 µM biotin in DMSO for 20 min at 37 °C, then immediately washed with ice cold PBS twice to stop the biotinylation reaction.

### mRNA extraction and qPCR

1 × 10^6^ cells were pelleted by centrifugation at 250 g, 4 °C for 5 min, and RNA extracted using the Isolate II RNA Mini kit (Qiagen), followed by cDNA conversion performed using the SuperScript III Reverse Transcriptase kit both according to manufacturer’s instructions. qPCR analysis was performed with 8 ng of cDNA using primers specific to ENO1 and the SensiMix SYBR Master Mix system under the following cycle conditions: 10 min 95 °C, 40 x cycles (30 s 95 °C, 30 s 62 °C, 30 s 72 °C, 15 s 95 °C), then repeat cycles with temperature increasing by 0.5 °C each cycle until 95 °C. Data was analysed to determine fold changes compared to the housekeeping genes UBC or 18 S, using the -∆∆Ct method [73]. Fold change was determined by dividing the mean starting quantity of MCF10AT, MCF10Ca1h or MCF10Ca1a samples by the mean starting quantity of MCF10A samples normalised to the mean of the housekeeping genes to determine the fold change gene expression between samples. Specific primer sequences are detailed in Supplementary Table 2.

### Protein extraction and Western blot analysis

Lysates were generated from twice washed cell pellets suspended in RIPA lysis buffer (50 µl per 1 × 10^6^ cells) at 4 °C for 10 min. Lysates were aspirated using a 26 G needle, transferred to chilled tubes, and centrifuged at max speed for 30 min at 4 °C. Supernatant was collected and left at -80 °C until use. Cell lysate concentration was determined using the BioRad Protein assay kit or D C (detergent compatible) Protein assay (both from Bio-Rad) according to manufacturer’s instructions.

Thawed lysates were subjected to SDS Polyacrylamide Gel Electrophoresis (SDS-PAGE) on 10% or 12% polyacrylamide gels at 90 V for 15 min, then 110 V for 1–2 h, in 1 X SDS running buffer. For mass spectrometry, immunoprecipitated samples were run on pre-cast 10% bis-tris polyacrylamide gels under the same voltage conditions. Proteins were transferred to nitrocellulose membrane at 90 V for 1 h at 4 °C, then blocked in 1% skim milk in PBST (PBS 0.1% Tween-20) blocking buffer, rocking overnight at 4 °C. Membranes were stained using primary antibodies against ENO1 (1:2000 in PBST), biotin (1:3000 in PBST), actin (1:500 in PBST) GFP (1:5000) and pan-phospho-serine (1:1000 in 1% BSA), then washed and incubated with fluorescent secondary antibodies (680-anti-rabbit and 800-anti-mouse) at 1:10000. All washes were performed in PBST at room temperature (RT), with a final rinse of PBS alone prior to imaging using a Li-Cor Odyssey scanner. Densitometric quantitation of bands normalised to actin loading controls was performed using ImageJ 1.53q software (NIH).

### Immunofluorescence

Cells pre-seeded and treated as specified were fixed with 4% PFA for 20 min at 37 °C, permeabilised with PBS/0.02% Triton-X-100 for 20 min and blocked with PBS/1% BSA overnight at 4 °C. Cells were incubated with anti-ENO1 (1:400), anti-V5 (1:400) or streptavidin-FITC (5 ug/ml) diluted in PBS/1% BSA for 1 h at RT. Following five PBS washes, cells were incubated with goat-anti-mouse or goat-anti-rabbit fluorescent secondary antibodies at 1:1000 for 1 h at RT in the dark. Coverslips were mounted using Prolong-gold antifade mounting reagent with DAPI (Thermo Fisher Scientific). Samples were left at RT in complete darkness after curing prior to imaging.

### Confocal Laser Scanning Microscopy (CLSM)

Fixed or live cells were imaged using an Olympus Fluoview FV1000 inverted confocal microscope using a 100X oil immersion lens (Monash Micro Imaging, Clayton). The nuclear to cytoplasmic fluorescence ratio (Fn/c) was determined as previously [74] using ImageJ 1.53q (NIH) to determine the nuclear (Fn) to cytoplasmic (Fc) fluorescence ratio (Fn/c) according to the formula: Fn/c = (Fn – Fb) / (Fc – Fb), where Fb is background due to autofluorescence. Cells with a Fn/c > 1 indicate nuclear localisation, where Fn/c < 1 indicates cytoplasmic localisation, and a Fn/c < 0.4 indicates nuclear exclusion. Fn/c ratios were calculated from samples with *n* > 30 cells analysed per sample.

### Purification of His_6_-tagged ENO1 proteins

Full length recombinant proteins ENO1 (wild-type, in plasmid pet14b) and ENO1-S419A (phospho-null mutant, also in pet14b) were expressed in Rosetta BL21(DE3) cells at 16 °C following induction at OD600nm = 0.8 with 1 mM isopropyl 1-thiol-β-D-galactopyranoside (IPTG; Astral Scientific). 18 h post-induction, bacteria were harvested by centrifugation and resuspended in lysis buffer containing 20 mM Tris-Cl or NaH_2_PO_4_ pH 8.0, 500 mM NaCl, 10% (v/v) glycerol, 0.5 mM TCEP, 10 mM imidazole, supplemented with 1 mg/mL lysozyme, and cOmplete protease inhibitor. Proteins were extracted by sonication and clarified by centrifugation, then applied to Ni-NTA Superflow resin (Qiagen). Resin was washed with lysis buffer three times, then His_6_-tagged-proteins were eluted with lysis buffer without lysozyme and containing 300 mM imidazole. Recombinant proteins were verified by SDS-PAGE and immunoblot. Fractions containing His_6_-ENO1 or His_6_-ENO1-S419A, were dialysed overnight to remove imidazole and concentrated by centrifugal filtration (Amicon Ultra-15 3 K MWCO) to 4 mg/ml and frozen at -80 °C. Lysis, washes, elutions and concentrating were performed on ice or at 4 °C.

### ENO1 activity assay

Samples containing MCF10A, MCF10AT, MCF10Ca1h and MCF10Ca1a cell lysate in extraction buffer, or purified ENO1 or ENO1-S419A in 1 x PBS buffer had enzyme activity determined by measuring NADH consumption using the ENO1 Human Activity Assay Kit (ab117994, Abcam), according to the manufacturer’s instructions. Relative activity of the samples was calculated against the activity of catalytically active ENO1 protein (Abcam; ab89248).

### Immunopurification and protein digestion for LC-MS

For the miniTurboID experiment, 15 cm dishes of MCF10Ca1h cells expressing V5-miniTurbo-ENO1 or V5-miniTurbo were treated with D4476 or DMSO, and biotin as in Supplementary Table 3. Cells were scraped and lysed as above. 300 µg of cell lysate per sample was incubated with 90 µl streptavidin magnetic beads in triplicate for 2 h at 4 °C, then washed 5 times with 50 mM TEAB. Samples on beads were reduced in 10 mM tris (2-carboxyethyl) phosphine (TCEP) made up in 50 mM ammonium bicarbonate (ABC) for 1 h at 37 °C. Proteins were then alkylated in darkness with 55 mM iodoacetamide in 50 mM ABC for 45 min at 37 °C. Samples were digested on bead in 25 ng/µl trypsin for 18 h at 37 °C. Peptides were recovered from beads by centrifugation, then freeze dried and rehydrated in MS loading buffer (2% ACN, 98% Water and 0.05% TFA).

### Liquid chromatography-mass spectrometry

Mass spectrometry was performed entirely at Bio21 Institute’s mass spectrometry and proteomics facility (MSPF). All samples were analysed by LC-MS using a Q-Exactive Plus mass spectrometer (Thermo Scientific) fitted with nanoflow reversed-phase-HPLC (Ultimate 3000 RSLC, Dionex). The nano-LC system was equipped with an Acclaim Pepmap nano-trap column (Dionex – C18, 100 Å, 75 μm × 2 cm) and an Acclaim Pepmap RSLC analytical column (Dionex – C18, 100 Å, 75 μm × 50 cm). For each LC-MS/MS experiment, 5 µl of the peptide mix was loaded onto the enrichment (trap) column at an isocratic flow of 5 µl/min of 3% ACN containing 0.1% formic acid (FA) for 6 min before the enrichment column was switched in-line with the analytical column. The eluents used for the LC were 0.1% v/v FA in water (solvent A) and 100% ACN/0.1% FA v/v (Solvent B). The gradient used was 2% B to 23% B for 29 min, 23% B to 40% B in 10 min, 40% B to 80% B in 5 min and maintained at 80% B for the final 5 min before equilibration for 10 min at 2% B prior to the next analysis. All spectra were acquired in positive mode with full scan MS spectra scanning from m/z 375–1400 at 70,000 resolutions with AGC target of 3e6 with a maximum accumulation time of 50 ms. The 15 most intense peptide ions with charge state ≥ 2–5 were isolated with an isolation window of 1.2 m/z and fragmented with a normalised collision energy of 30 at 17,500 resolution with AGC target of 5e4 with a maximum accumulation time of 50 ms. Dynamic exclusion was activated for 30 s.

### Proteomics analysis

MiniTurboID analysis was carried out similar to Formosa et al. [75]. Raw files were first analysed in MaxQuant as described, but with “label free quantitation” set to “LFQ” and “match between runs” disabled. Data output was then analysed in Perseus software, where proteins group LFQ intensities were Log2 transformed. Values listed as being ‘Only identified by site’, ‘Reverse’ or ‘Contaminants’ were removed from the dataset. Experimental groups were assigned to each set of triplicates and the number of valid values for row group calculated. For each experiment rows having less than three valid values in the miniTurbo-ENO1 expressing D4476 treated group were removed and the missing values in the relevant control group imputed to values consistent with the limit of detection. A modified two-sided Student’s t test based on permutation-based FDR statistics [76] was performed between the two groups. The negative logarithmic p-values were plotted against the differences between the Log2 means for the experimental D4476 treated and control DMSO treated MCF10Ca1h expressing MiniTurbo-ENO1 groups. MCF10Ca1h samples expressing MiniTurbo alone or samples that were not biotin treated to induce proximity labelling were used to refine genuine MiniTurbo-ENO1 interacting proteins from endogenously biotinylated or non-specific proteins. Gene Ontology (GO) terms were clustered using the PANTHER online platform [77] to annotate function and interacting pathways for enriched or depleted protein groups between samples [78].

The confidence score was calculated using the posterior error probability (PEP) which is derived from the target and decoy distributions of the protein group scores, where confidence = 1-PEP. For a confidence score of 95% it is expected that 5% of peptide spectral matches (PSMs) are false positives based on the number of matches identified before decoy sequence hits.

### Statistical analysis

Statistical analysis was performed in GraphPad Prism 9.3.1 unless otherwise specified. Data was analysed by unpaired t-test with Welch correction. Datasets with > 2 samples were analysed for significant differences using Brown-Forsyth and Welch ANOVA, comparisons to control samples were analysed by unpaired T-test with Welch correction, without correction for multiple comparisons. 2-tailed p-values were considered statistically significant when *p* < 0.05.

### Electronic supplementary material

Below is the link to the electronic supplementary material.

Supplementary Material 1

Supplementary Material 2

## Data Availability

The results shown in Fig. 3A are in part based upon publicly available data generated by the TCGA Research Network: https://www.cancer.gov/tcga. All other data is available from the authors upon reasonable request.

## References

[1] Yang R, Li Y, Wang H, Qin T, Yin X, Ma X (2022). Therapeutic progress and challenges for triple negative breast cancer: targeted therapy and immunotherapy. Mol Biomed.

[2] Won KA, Spruck C (2020). Triple–negative breast cancer therapy: current and future perspectives (review). Int J Oncol.

[3] Oualla K, El-Zawahry HM, Arun B, Reuben JM, Woodward WA, Gamal El-Din H (2017). Novel therapeutic strategies in the treatment of triple-negative breast cancer. Ther Adv Med Oncol.

[4] Bianchini G, Balko JM, Mayer IA, Sanders ME, Gianni L (2016). Triple-negative breast cancer: challenges and opportunities of a heterogeneous disease. Nat Rev Clin Oncol.

[5] Hippner M, Majkowski M, Biecek P, Szkudlarek T, Simiczyjew A, Pieniazek M, et al. Alpha-Enolase (ENO1) Correlates with Invasiveness of Cutaneous Melanoma&mdash;An In Vitro and a Clinical Study. Diagnostics. 2022;12(2):254. 10.3390/diagnostics12020254 PMC8871300 35204345

[6] Mittal S, Kaur H, Gautam N, Mantha AK (2017). Biosensors for breast cancer diagnosis: a review of bioreceptors, biotransducers and signal amplification strategies. Biosens Bioelectron.

[7] Tyanova S, Albrechtsen R, Kronqvist P, Cox J, Mann M, Geiger T (2016). Proteomic maps of breast cancer subtypes. Nat Commun.

[8] Lai YW, Hsu WJ, Lee WY, Chen CH, Tsai YH, Dai JZ et al. Prognostic value of a Glycolytic Signature and its regulation by Y-Box-binding protein 1 in Triple-negative breast Cancer. Cells. 2021;10(8). 10.3390/cells10081890 PMC8392807 34440660

[9] Edwards YH, Grootegoed JA (1983). A sperm-specific enolase. J Reprod Fertil.

[10] Nakamura N, Dai Q, Williams J, Goulding EH, Willis WD, Brown PR (2013). Disruption of a spermatogenic cell-specific mouse enolase 4 (eno4) gene causes sperm structural defects and male infertility. Biol Reprod.

[11] Ji H, Wang J, Guo J, Li Y, Lian S, Guo W (2016). Progress in the biological function of alpha-enolase. Anim Nutr.

[12] Feo S, Arcuri D, Piddini E, Passantino R, Giallongo A (2000). ENO1 gene product binds to the c-myc promoter and acts as a transcriptional repressor: relationship with myc promoter-binding protein 1 (MBP-1). FEBS Lett.

[13] Subramanian A, Miller DM (2000). Structural analysis of alpha-enolase. Mapping the functional domains involved in down-regulation of the c-myc protooncogene. J Biol Chem.

[14] Sedoris KC, Thomas SD, Miller DM (2010). Hypoxia induces differential translation of enolase/MBP-1. BMC Cancer.

[15] Sun X, Wang M, Wang M, Yu X, Guo J, Sun T (2020). Metabolic reprogramming in Triple-negative breast Cancer. Front Oncol.

[16] Capello M, Ferri-Borgogno S, Riganti C, Chattaragada MS, Principe M, Roux C (2016). Targeting the Warburg effect in cancer cells through ENO1 knockdown rescues oxidative phosphorylation and induces growth arrest. Oncotarget.

[17] Qiao G, Wu A, Chen X, Tian Y, Lin X (2021). Enolase 1, a moonlighting protein, as a potential target for Cancer Treatment. Int J Biol Sci.

[18] Schofield L, Lincz LF, Skelding KA (2020). Unlikely role of glycolytic enzyme α-enolase in cancer metastasis and its potential as a prognostic biomarker. J Cancer Metastasis Treat.

[19] Zang HY, Gong LG, Li SY, Hao JG (2020). Inhibition of α-enolase affects the biological activity of breast cancer cells by attenuating PI3K/Akt signaling pathway. Eur Rev Med Pharmacol Sci.

[20] Tu SH, Chang CC, Chen CS, Tam KW, Wang YJ, Lee CH (2010). Increased expression of enolase alpha in human breast cancer confers tamoxifen resistance in human breast cancer cells. Breast Cancer Res Treat.

[21] Didiasova M, Schaefer L, Wygrecka M (2019). When place matters: shuttling of Enolase-1 Across Cellular compartments. Front Cell Dev Biology.

[22] Zhu X, Miao X, Wu Y, Li C, Guo Y, Liu Y (2015). ENO1 promotes tumor proliferation and cell adhesion mediated drug resistance (CAM-DR) in Non-hodgkin’s lymphomas. Exp Cell Res.

[23] Georges E, Bonneau AM, Prinos P (2011). RNAi-mediated knockdown of α-enolase increases the sensitivity of tumor cells to antitubulin chemotherapeutics. Int J Biochem Mol Biol.

[24] Capello M, Caorsi C, Bogantes Hernandez PJ, Dametto E, Bertinetto FE, Magistroni P (2015). Phosphorylated alpha-enolase induces autoantibodies in HLA-DR8 pancreatic cancer patients and triggers HLA-DR8 restricted T-cell activation. Immunol Lett.

[25] López-Alemany R, Longstaff C, Hawley S, Mirshahi M, Fábregas P, Jardí M (2003). Inhibition of cell surface mediated plasminogen activation by a monoclonal antibody against alpha-enolase. Am J Hematol.

[26] Cappello P, Rolla S, Chiarle R, Principe M, Cavallo F, Perconti G (2013). Vaccination with ENO1 DNA prolongs survival of genetically engineered mice with pancreatic cancer. Gastroenterology.

[27] Almaguel FA, Sanchez TW, Ortiz-Hernandez GL, Casiano CA (2020). Alpha-Enolase: emerging Tumor-Associated Antigen, Cancer Biomarker, and Oncotherapeutic Target. Front Genet.

[28] Huang CK, Sun Y, Lv L, Ping Y (2022). ENO1 and Cancer. Mol Ther Oncolytics.

[29] Ceruti P, Principe M, Capello M, Cappello P, Novelli F (2013). Three are better than one: plasminogen receptors as cancer theranostic targets. Experimental Hematol Oncol.

[30] Gao S, Li H, Cai Y, Ye JT, Liu ZP, Lu J (2014). Mitochondrial binding of α-enolase stabilizes mitochondrial membrane: its role in doxorubicin-induced cardiomyocyte apoptosis. Arch Biochem Biophys.

[31] Henderson MC, Azorsa DO (2012). The genomic and proteomic content of cancer cell-derived exosomes. Front Oncol.

[32] Didiasova M, Zakrzewicz D, Magdolen V, Nagaraj C, Bálint Z, Rohde M (2015). STIM1/ORAI1-mediated Ca2 + influx regulates Enolase-1 exteriorization. J Biol Chem.

[33] Cancemi P, Buttacavoli M, Roz E, Feo S. Expression of alpha-enolase (ENO1), myc promoter-binding Protein-1 (MBP-1) and Matrix metalloproteinases (MMP-2 and MMP-9) reflect the nature and aggressiveness of breast tumors. Int J Mol Sci. 2019;20(16). 10.3390/ijms20163952 PMC6720302 31416219

[34] Czogalla B, Partenheimer A, Badmann S, Schmoeckel E, Mayr D, Kolben T (2021). Nuclear Enolase-1/ MBP-1 expression and its association with the wnt signaling in epithelial ovarian cancer. Transl Oncol.

[35] Lo Presti M, Ferro A, Contino F, Mazzarella C, Sbacchi S, Roz E (2010). Myc promoter-binding Protein-1 (MBP-1) is a novel potential prognostic marker in invasive ductal breast carcinoma. PLoS ONE.

[36] Prieto G, Fullaondo A, Rodríguez JA (2016). Proteome-wide search for functional motifs altered in tumors: prediction of nuclear export signals inactivated by cancer-related mutations. Sci Rep.

[37] Conforti F, Wang Y, Rodriguez JA, Alberobello AT, Zhang Y-W, Giaccone G (2015). Molecular pathways: Anticancer Activity by Inhibition of Nucleocytoplasmic shuttling. Clin Cancer Res.

[38] Mahipal A, Malafa M (2016). Importins and exportins as therapeutic targets in cancer. Pharmacol Ther.

[39] Mouveaux T, Oria G, Werkmeister E, Slomianny C, Fox BA, Bzik DJ (2014). Nuclear glycolytic enzyme enolase of Toxoplasma Gondii functions as a Transcriptional Regulator. PLoS ONE.

[40] Pal-Bhowmick I, Vora HK, Jarori GK (2007). Sub-cellular localization and post-translational modifications of the Plasmodium Yoelii enolase suggest moonlighting functions. Malar J.

[41] Tomaino B, Cappello P, Capello M, Fredolini C, Sperduti I, Migliorini P (2011). Circulating autoantibodies to phosphorylated α-Enolase are a Hallmark of Pancreatic Cancer. J Proteome Res.

[42] Zakrzewicz D, Didiasova M, Krüger M, Giaimo BD, Borggrefe T, Mieth M, et al (2018). Protein arginine methyltransferase 5 mediates enolase-1 cell surface trafficking in human lung adenocarcinoma cells. Biochim et Biophys Acta (BBA) - Mol Basis Disease.

[43] Ou B, Liu Y, Yang X, Xu X, Yan Y, Zhang J (2021). C5aR1-positive neutrophils promote breast cancer glycolysis through WTAP-dependent m6A methylation of ENO1. Cell Death Dis.

[44] Zhan P, Wang Y, Zhao S, Liu C, Wang Y, Wen M (2015). FBXW7 negatively regulates ENO1 expression and function in colorectal cancer. Lab Invest.

[45] Phan L, Chou P-C, Velazquez-Torres G, Samudio I, Parreno K, Huang Y (2015). The cell cycle regulator 14-3-3σ opposes and reverses cancer metabolic reprogramming. Nat Commun.

[46] Zheng F, Jang W-C, Fung FKC, Lo ACY, Wong IYH (2016). Up-Regulation of ENO1 by HIF-1α in retinal pigment epithelial cells after hypoxic challenge is not involved in the regulation of VEGF secretion. PLoS ONE.

[47] Zheng R, Yao Q, Li X, Xu B (2019). Long noncoding ribonucleic acid SNHG18 promotes glioma cell motility via disruption of α-Enolase nucleocytoplasmic transport. Front Genet.

[48] Yu S, Li N, Huang Z, Chen R, Yi P, Kang R (2018). A novel lncRNA, TCONS_00006195, represses hepatocellular carcinoma progression by inhibiting enzymatic activity of ENO1. Cell Death Dis.

[49] Shevade S, Jindal N, Dutta S, Jarori GK (2013). Food Vacuole Associated Enolase in Plasmodium undergoes multiple post-translational modifications: evidence for atypical ubiquitination. PLoS ONE.

[50] Li Terytty Y, Sun Y, Liang Y, Liu Q, Shi Y, Zhang C-S (2016). ULK1/2 constitute a Bifurcate Node Controlling glucose metabolic fluxes in Addition to Autophagy. Mol Cell.

[51] Wagstaff KM, Jans DA (2009). Importins and Beyond: Non-conventional Nuclear Transport mechanisms. Traffic.

[52] Hutten S, Kehlenbach RH (2007). CRM1-mediated nuclear export: to the pore and beyond. Trends Cell Biol.

[53] Poon IKH, Jans DA (2005). Regulation of Nuclear Transport: Central Role in Development and Transformation?. Traffic.

[54] Zhou W, Capello M, Fredolini C, Piemonti L, Liotta LA, Novelli F (2010). Mass Spectrometry Analysis of the post-translational modifications of α-Enolase from pancreatic ductal adenocarcinoma cells. J Proteome Res.

[55] Li Y, Kong X, Wang Z, Xuan L (2022). Recent advances of transcriptomics and proteomics in triple-negative breast cancer prognosis assessment. J Cell Mol Med.

[56] Comprehensive molecular portraits (2012). Of human breast tumours. Nature.

[57] Mertins P, Mani DR, Ruggles KV, Gillette MA, Clauser KR, Wang P (2016). Proteogenomics connects somatic mutations to signalling in breast cancer. Nature.

[58] Hornbeck PV, Zhang B, Murray B, Kornhauser JM, Latham V, Skrzypek E (2015). PhosphoSitePlus, 2014: mutations, PTMs and recalibrations. Nucleic Acids Res.

[59] Rena G, Bain J, Elliott M, Cohen P (2004). D4476, a cell-permeant inhibitor of CK1, suppresses the site-specific phosphorylation and nuclear exclusion of FOXO1a. EMBO Rep.

[60] Branon TC, Bosch JA, Sanchez AD, Udeshi ND, Svinkina T, Carr SA (2018). Efficient proximity labeling in living cells and organisms with TurboID. Nat Biotechnol.

[61] Tytgat HL, Schoofs G, Driesen M, Proost P, Van Damme EJ, Vanderleyden J (2015). Endogenous biotin-binding proteins: an overlooked factor causing false positives in streptavidin-based protein detection. Microb Biotechnol.

[62] Zhang J, Li H, Miao L, Ding J (2020). Silencing of ENO1 inhibits the proliferation, migration and invasion of human breast cancer cells. J BUON.

[63] Capello M, Ferri-Borgogno S, Cappello P, Novelli F (2011). α-enolase: a promising therapeutic and diagnostic tumor target. FEBS J.

[64] Siri M, Behrouj H, Dastghaib S, Zamani M, Likus W, Rezaie S (2021). Casein kinase-1-Alpha inhibitor (D4476) sensitizes microsatellite instable colorectal Cancer cells to 5-Fluorouracil via Authophagy Flux Inhibition. Arch Immunol Ther Exp.

[65] Kishimoto N, Yamamoto K, Iga N, Kirihara C, Abe T, Takamune N (2020). Alpha-enolase in viral target cells suppresses the human immunodeficiency virus type 1 integration. Retrovirology.

[66] Kuusisto HV, Wagstaff KM, Alvisi G, Jans DA (2008). The C-terminus of apoptin represents a unique tumor cell-enhanced nuclear targeting module. Int J Cancer.

[67] Poon IKH, Oro C, Dias MM, Zhang J, Jans DA (2005). Apoptin Nuclear Accumulation is modulated by a CRM1-Recognized Nuclear Export Signal that is active in normal but not in Tumor cells. Cancer Res.

[68] Rosenberg LH, Lafitte M, Quereda V, Grant W, Chen W, Bibian M (2015). Therapeutic targeting of casein kinase 1δ in breast cancer. Sci Transl Med.

[69] Bar I, Merhi A, Larbanoix L, Constant M, Haussy S, Laurent S (2018). Silencing of casein kinase 1 delta reduces migration and metastasis of triple negative breast cancer cells. Oncotarget.

[70] Mazzoldi EL, Pastò A, Ceppelli E, Pilotto G, Barbieri V, Amadori A (2019). Casein Kinase 1 Delta regulates cell proliferation, response to Chemotherapy and Migration in Human Ovarian Cancer cells. Front Oncol.

[71] Santner S, Dawson P, Tait L, Soule H, Eliason J, Mohamed A (2001). Malignant MCF10CA1 cell lines derived from Premalignant Human breast epithelial MCF10AT cells. Breast Cancer Res Treat.

[72] Kuusisto HV, Jans DA (2015). Hyper-dependence of breast cancer cell types on the nuclear transporter importin β1. Biochimica et Biophysica Acta (BBA). Mol Cell Res.

[73] Schmittgen TD, Livak KJ (2008). Analyzing real-time PCR data by the comparative C(T) method. Nat Protoc.

[74] Gajewska KA, Lescesen H, Ramialison M, Wagstaff KM, Jans DA (2021). Nuclear transporter Importin-13 plays a key role in the oxidative stress transcriptional response. Nat Commun.

[75] Formosa LE, Muellner-Wong L, Reljic B, Sharpe AJ, Jackson TD, Beilharz TH et al. Dissecting the roles of mitochondrial complex I Intermediate Assembly Complex factors in the Biogenesis of Complex I. Cell Rep. 2020;31(3). 10.1016/j.celrep.2020.107541 32320651

[76] Tyanova S, Temu T, Sinitcyn P, Carlson A, Hein MY, Geiger T (2016). The Perseus computational platform for comprehensive analysis of (prote)omics data. Nat Methods.

[77] Thomas PD, Ebert D, Muruganujan A, Mushayahama T, Albou L-P, Mi H (2022). PANTHER: making genome-scale phylogenetics accessible to all. Protein Sci.

[78] Mi H, Thomas P (2009). PANTHER pathway: an ontology-based pathway database coupled with data analysis tools. Methods Mol Biol.

