# Supplementary material for: Tumour-specific phosphorylation of serine 419 drives alpha-enolase (ENO1) nuclear export in triple negative breast cancer progression
Source: Cell Biosci. 2024 Jun 7;14:74. doi: 10.1186/s13578-024-01249-x (PMC11157870; doi:10.1186/s13578-024-01249-x)
Supplement: Supplementary file 2 — Supplementary Material 2 [file 13578_2024_1249_MOESM2_ESM.pdf]

## Supplementary Figures

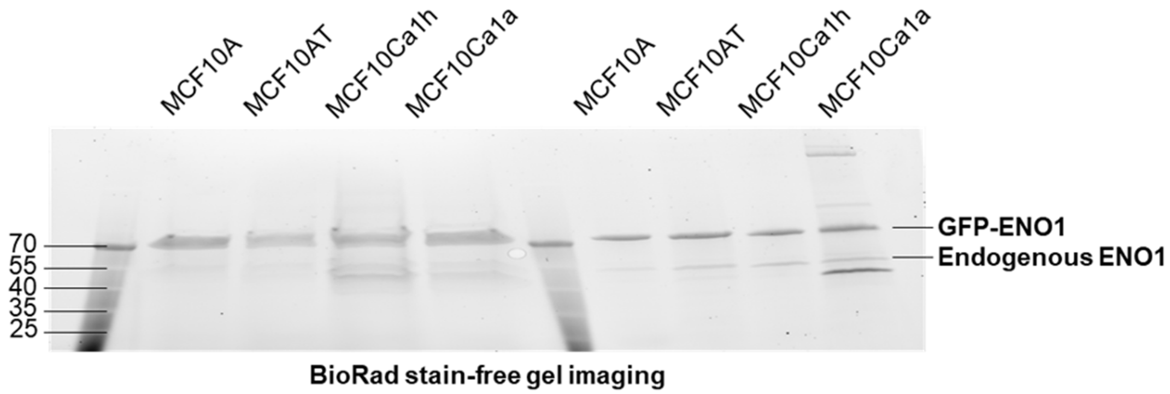


**Supplementary Figure S1. Total protein visualisation on gel indicating immunopurified GFP-ENO1 protein bands prior to mass spectrometry analysis.** Proteins bound to GFP-trap beads isolated from GFP-ENO1 transfected MCF10 cells were separated by SDS-PAGE and total protein was imaged using BioRad stain-free imaging technology. Bands of the correct molecular weight of GFP-ENO1 (~70 kDa) and untagged endogenous ENO1 (~47 kDa) (expected to be immunopurified due to homodimerisation) were cut out for subsequent mass spectrometry analysis. PageRuler molecular weight standards are indicated in kDa.

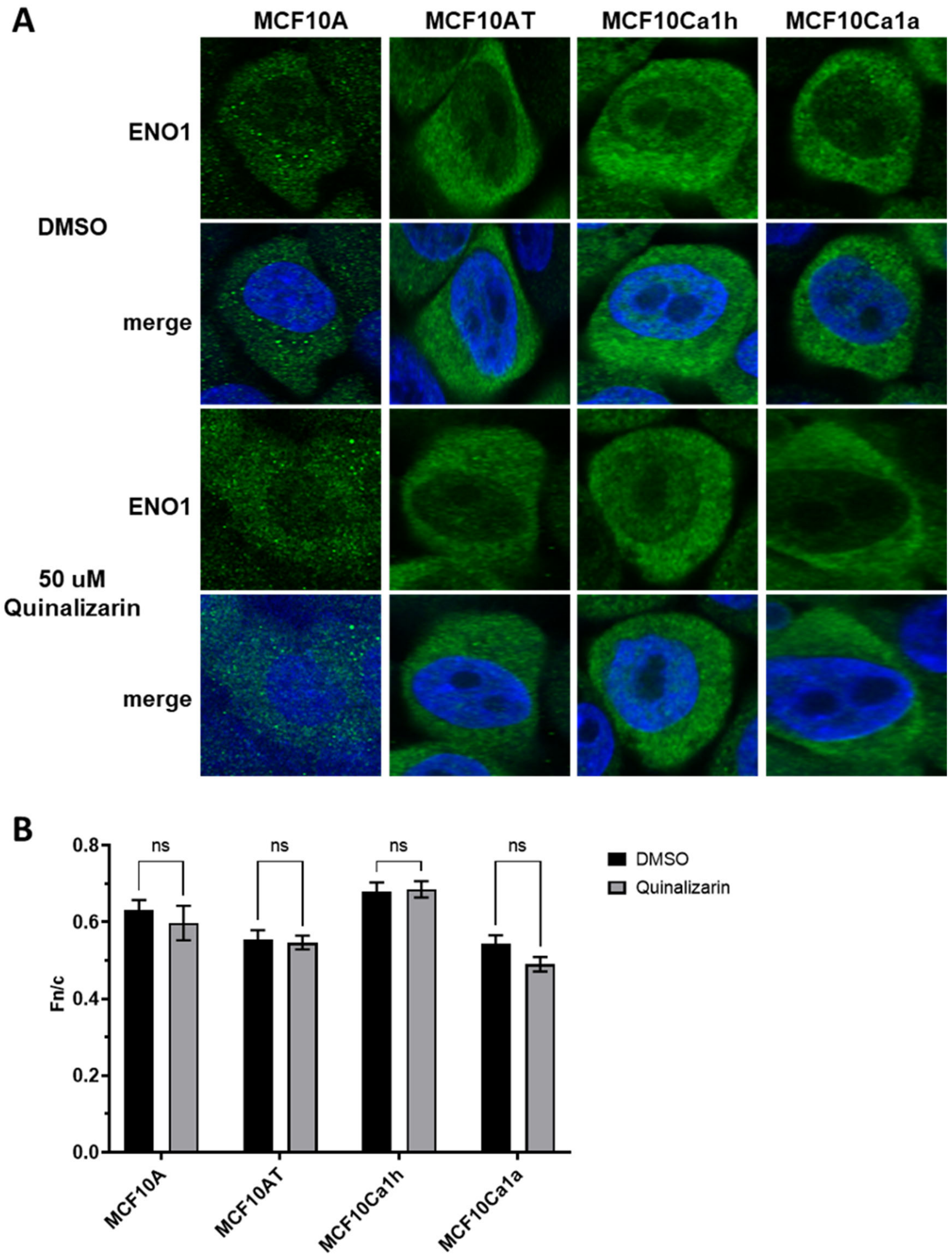


**Supplementary Figure S2. Casein kinase 2 inhibition with Quinalizarin does not alter ENO1 nuclear accumulation in MCF10 cell lines.** (A) Representative images of MCF10 TNBC cell lines treated with 50 μM casein kinase 2 inhibitor Quinalizarin or DMSO for 3 h, fixed and stained with anti-ENO1 antibodies and imaged by CLSM. (B) Images such as those in (A) were analysed to determine Fn/c ratio as previously. Results represent mean Fn/c ± SEM of a single typical experiment from a series of 3 similar experiments. ns = non-significant compared to DMSO treated cell line.

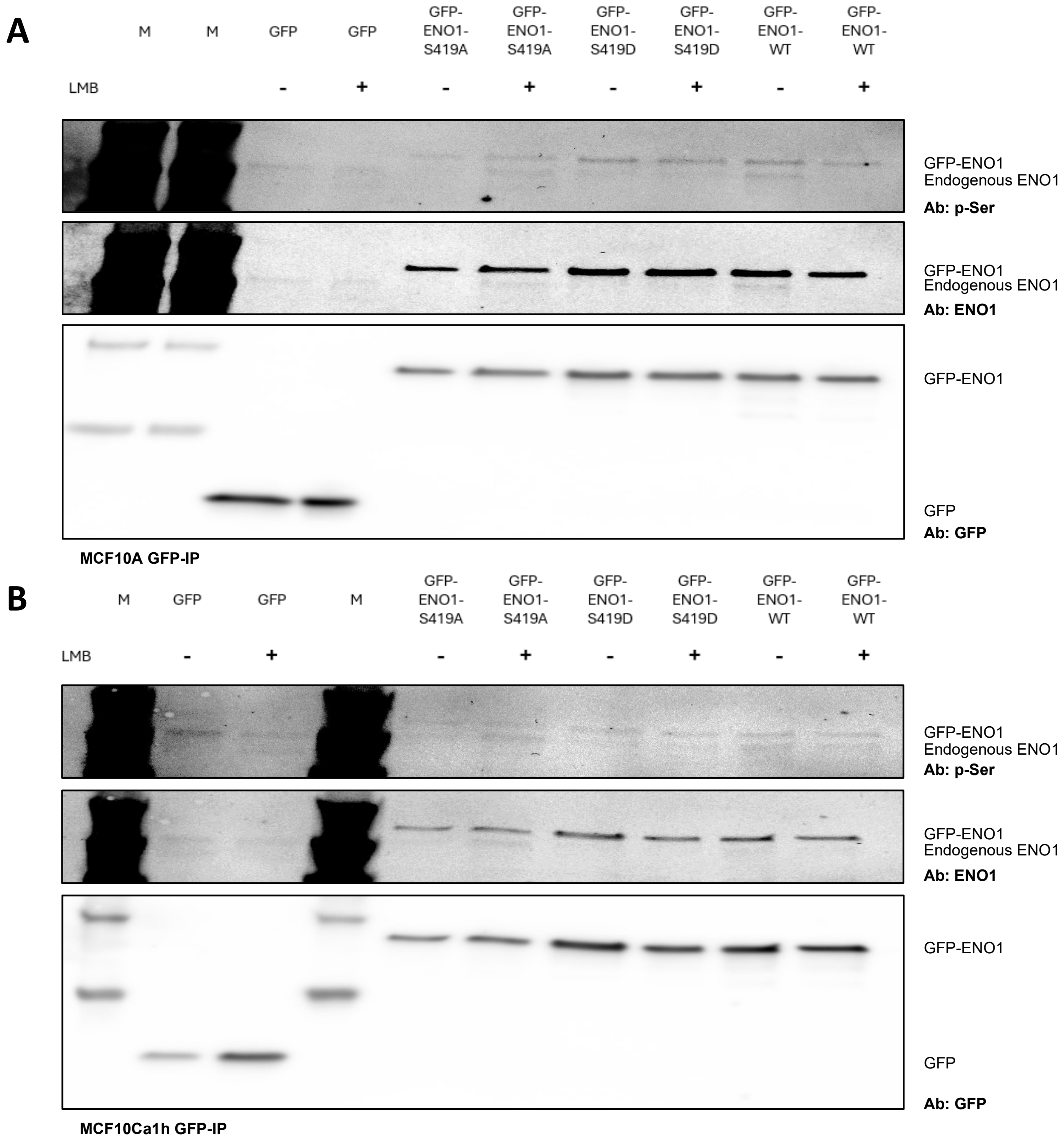


**Supplementary Figure S3. LMB treatment and CMR1-mediated nuclear export blockage does not alter the phosphorylation state of GFP-ENO1-S419 mutants.** Western blot of GFP-ENO1 and phospho-serine expression in MCF10A and MCF10Ca1h TNBC cell lines. GFP-trap immunoprecipitations of the indicated MCF10 cell lines were subjected to western blot and probed with anti-phospho-serine, ENO1, and GFP antibodies. (A) MCF10A cells transfected with GFP-S419 mutants were treated with LMB and GFP-ENO1 proteins were immunoprecipitated to determine phospho-serine presence. (B) MCF10Ca1h cells transfected with GFP-S419 mutants were treated with LMB and GFP-ENO1 proteins were immunoprecipitated to determine phospho-serine presence.

In both immunoprecipitations (IPs) there is evident endogenous ENO1 pulldown along with GFP-tagged ENO1, this is due to the known dimerization of ENO1, which also appear faintly in the anti-phospho-serine and ENO1 probed blots. LMB – leptomycin B, p-Ser – global anti-phospho-serine-antibody. Result representative of a single typical experiment from a series of 2 similar experiments.

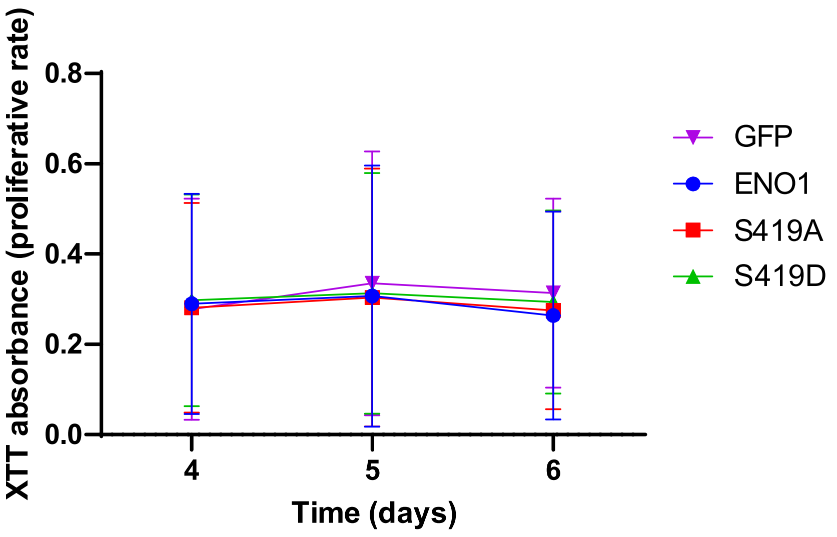


**Supplementary Figure S4. Expression of ENO1 S419 point mutants has no effect on proliferation and shows mild toxicity to MCF10A non-tumour cells.** MCF10A non-tumour cells were transfected with GFP, GFP-ENO1, GFP-ENO1-S419A or –S419D on day 3 timepoint after seeding into 96-well tissue culture plates (XTT absorbance not measured until day 4). Cell proliferation rate was measured using XTT assay, over 3 biological replicate experiments we observed no reproducible growth trends between treatments and slight cellular toxicity following expression over 4-6 days post seeding. Leading us to conclude that introduction of ENO1 point mutant expression was not possible with our chosen transfection approach and non-tumour cell lines. We observed minimal toxicity in MCF10Ca1h tumour cells in a similar experiment however again no reproducible trends were observed, and we could not discern between the action of the endogenous ENO1 and our GFP-expression constructs in tumour cells.

The Microsoft Excel file containing Supplementary Table 1 can be accessed at the following link below

Supplementary Table 1:
https://docs.google.com/spreadsheets/d/1BYV5rg6KQq9g6MlGfhMR3xm8mnMsHQCA/edit?usp=sharing&ouid=108983312601833825244&rtpof=true&sd=true

Supplementary Table 2 – primer sequences for qPCR

| Primer Target | Forward Primer | Reverse Primer |
|---|---|---|
| ENO1 | AAGGTCTCTTCAGAGCTGC | GTTCAGTTTCTTGCTAACCAGG |
| UBC | ATTTGGGTCGCGGTTCTTG | TGCCTTGACATTCTCGATGGT |
| 18S | TCCCCCAACTTCTTAGAGG | CTTATGACCCGCACTTACTG |

Supplementary Table 3 – ENO1-miniTurboID samples and controls

| **Cell line** | **Plasmid** | **D4476 treatment** | **Biotin treated to induce proximity labelling** | **Purpose** |
|---|---|---|---|---|
| MCF10Ca1h | miniTurbo | + | - | Background control |
| MCF10Ca1h | miniTurbo | - | 50 μM biotin | Non-specific interaction control |
| MCF10Ca1h | miniTurbo | + | 50 μM biotin | Non-specific interaction control |
| MCF10Ca1h | miniTurbo-ENO1 | + | - | Background control |
| MCF10Ca1h | miniTurbo-ENO1 | - | 50 μM biotin | Wildtype comparison |
| MCF10Ca1h | miniTurbo-ENO1 | + | 50 μM biotin | Experimental variable |
